# Examining temporal changes in model-optimized parameters using longitudinal hemodynamic measurements

**DOI:** 10.1186/s12938-024-01242-y

**Published:** 2024-07-10

**Authors:** Nikolai L. Bjørdalsbakke, Jacob Sturdy, Ulrik Wisløff, Leif R. Hellevik

**Affiliations:** 1 Department of Structural Engineering, Norwegian University of Science and Technology (NTNU), Richard Birkelandsvei 1A, Trondheim, 7491 Norway; 2 Cardiac Exercise Research Group at the Department of Circulation and Medical Imaging, Norwegian University of Science and Technology (NTNU), Prinsesse Kristinas gate 3, Trondheim, 7491 Norway

## Abstract

**Background:**

We previously applied hemodynamic data to personalize a mathematical model of the circulation expressed as physically interpretable parameters. The aim of this study was to identify patterns in the data that could potentially explain the estimated parameter changes. This included investigating whether the parameters could be used to track the effect of physical activity on high blood pressure. Clinical trials have repeatedly detected beneficial changes in blood pressure after physical activity and uncovered changes in lower level phenotypes (such as stiffened or high-resistance blood vessels). These phenotypes can be characterized by parameters describing the mechanical properties of the circulatory system. These parameters can be incorporated in and contextualized by physics-based cardiovascular models of the circulation, which in combination can become tools for monitoring cardiovascular disease progression and management in the future.

**Methods:**

Closed-loop and open-loop models of the left ventricle and systemic circulation were previously optimized to data from a pilot study with a 12-week exercise intervention period. Basal characteristics and hemodynamic data such as blood pressure in the carotid, brachial and finger arteries, as well as left-ventricular outflow tract flow traces were collected in the trial. Model parameters estimated for measurements made on separate days during the trial were used to compute parameter changes for total peripheral resistance, systemic arterial compliance, and maximal left-ventricular elastance. We compared the changes in these cardiovascular model-based estimates to changes from more conventional estimates made without the use of physics-based models by correlation analysis. Additionally, ordinary linear regression and linear mixed-effects models were applied to determine the most informative measurements for the selected parameters. We applied maximal aerobic capacity (measured as $\text{VO2max}$) data to examine if exercise had any impact on parameters through regression analysis and case studies.

**Results and conclusions:**

Parameter changes in arterial parameters estimated using the cardiovascular models correlated moderately well with conventional estimates. Estimates based on carotid pressure waveforms gave higher correlations (0.59 and above when p$<0.05$) than those for finger arterial pressure. Parameter changes over the 12-week study duration were of similar magnitude when compared to short-term changes after a bout of intensive exercise in the same parameters. The short-term changes were computed from measurements made immediately before and 24 h after a cardiopulmonary exercise test used to measure $\text{VO2max}$. Regression analysis indicated that changes in $\text{VO2max}$ did not account for any substantial amount of variability in total peripheral resistance, systemic arterial compliance, or maximal left-ventricular elastance. On the contrary, changes in stroke volume contributed to far more explained variability. The results suggest that more research is required to be able to accurately track exercise-induced changes in the vasculature for people with pre-hypertension and hypertension using lumped-parameter models.

## Introduction

Cardiovascular (CV) disease is a leading cause of loss of quality of life and premature death worldwide [1]. Disease progression is usually slow, and it may take years before detectable symptoms appear. Although it is possible to monitor biological and behavioral risk factors at regular clinical visits, these measures may not provide sufficient insight into the underlying mechanisms contributing to the disease. In the case of essential hypertension, a persistently elevated blood pressure without an identifiable medical cause, we envision that monitoring the underlying hemodynamics may improve early detection and intervention in primary prevention of CV disease [2]. By hemodynamics we refer to for example measurements of blood pressure and flow, such as systolic and diastolic brachial pressure, and cardiac output (CO). We believe that this can in part be achieved by application of personalized physics-based CV models. In the clinic we envision that one can detect sustained parameter changes in directions consistent with parameter sets which are found in elevated blood pressure at an early stage. Additionally, this could pinpoint which part of the circulation is remodeling and give health care practitioners more data to base decisions upon. Furthermore, observing parameter changes during therapy may give more detailed knowledge about which parameters the therapy successfully impacts in order to alleviate hypertension, and which parameters do not respond, and could possibly be targeted by other means. This is also based on a hypothesis that manifestations of the underlying causes of hypertension are sufficiently represented by these parameters. As the etiology of hypertension is not yet fully understood this is a working hypothesis.

Physics-based CV models have already been applied to predict the outcome of specific interventions [3–6]. Some of these studies focused on interventions which can be made rapidly by invasive procedures or medical treatments with changes expected to take effect almost immediately. Other studies focused directly on the post-intervention hemodynamics rather than the change in parameters themselves. In such contexts the parameter change can be prescribed according to what is altered during an invasive procedure or treatment and its development is not necessarily considered an interesting outcome in itself. Some examples such as work by Audebert et al. and by Gerringer et al. focused on a specific parameter during disease progression [7, 8]. Personalized CV models which predict the development of parameters given different stimuli can be valuable clinical tools and provide more detailed information about response to treatment beyond the measurable hemodynamics alone. For example, models could potentially give more insight into disease etiology than conventional parameter estimates alone, by providing continuous updates about lower level phenotypes normally not easily measurable. For this to be useful, parameter estimates and their changes must be reliable and interpretable in a clinical context. By conventional estimates, we refer to estimates made by methods that are algebraic in nature and not dependent upon models of the CV system. Here, we focus on parameters estimated for two lumped-parameter models and compare the results to their conventional estimates of similar parameters from the same hemodynamic measurements.

In this work, we treat the mechanical model parameters as the quantities of interest and use these as proxies for observing changes in low-level phenotypes in the progression of CV disease, such as arterial stiffening, change in vascular tone and altered cardiac hemodynamics. Regular physical activity is recommended in prevention and management of hypertension [9]. However, the effect depends on the duration, frequency, and intensity of exercise. Our hypothesis is that CV remodeling can be sufficiently represented by mechanical parameters which reflect the exercise-induced changes in hemodynamics.

Changes in habitual exercise have been observed to produce changes throughout the CV systems. A meta-analysis by Fagard et al. concluded that aerobic exercise lowers blood pressure and systemic vascular resistance in a mixed population with both normal and high blood pressure [10]. Molmen-Hansen et al. observed a significant reduction of total peripheral resistance in patients with hypertension undergoing aerobic interval training [11]. Ashor et al. compared studies with exercise interventions lasting between 8–26 weeks in a meta-review and reported reduced arterial stiffness estimated via pulse wave velocity (PWV) in individuals with high normal blood pressure and hypertension after aerobic exercise [12]. In contrast, Montero et al. found reduced arterial stiffness in adults with high normal blood pressure and hypertension only in studies with an exercise intervention longer than 12 weeks or where the change could be associated with a large reduction in systolic blood pressure [13]. PWV is not equivalent to the arterial compliance parameters often used in lumped-parameter models, but is related to the structural and material properties influencing arterial compliance. Changes in PWV thus suggest that exercise can affect arterial wall properties. The resting ventricular function is also affected by exercise. Molmen-Hansen also found changes in multiple markers related to ventricular contractility after aerobic interval training, such as ejection fraction (EF) and peak velocity of the tricuspid valve annulus in systole [11]. By studying rats after periods of training and detraining, Oláh et al. observed increase in the end-systolic left-ventricular elastance after 12 weeks of exercise [14]. Hence, we expect that vascular properties can change as an effect of regular physical activity.

An exercise motivation trial conducted at the Norwegian University of Science and Technology in 2019–2020 monitored the hemodynamics of participants three times during the 12-week intervention period. The trial was originally designed to examine the effect of using Personal Activity Intelligence (PAI) score as an exercise motivator and its effect on blood pressure. PAI score is the output of a mathematical model considering an individual’s heart rate history to compute an easily understandable personalized metric of physical activity [15, 16]. Using data from this trial, Bjørdalsbakke et al. personalized two simple CV models of the systemic circulation and analyzed the variability in parameter estimates due to personalization method as well as variation in the population [17]. The chosen personalization method was an ensemble method based on local non-linear optimization producing multiple parameter estimates to find an averaged solution after filtering out the worst estimates. Consequently, the choice of method introduced some variability to the parameter estimates themselves. Whether identified changes in parameters were primarily caused by the optimization procedure, the exercise intervention, or simply day-to-day variation in hemodynamics remained undetermined. Hence, the parameter changes during the intervention period and their explanation were the main focus of this manuscript. One way to approach this was to examine if identified patterns and trends were consistent with previous knowledge about the dependence on these parameters to exercise adjacent indices. The analysis was undertaken by focusing on the arterial parameters of total peripheral resistance, systemic arterial compliance, as well as maximal left-ventricular elastance. We are careful not to draw conclusions about causality from this analysis as both the data set is meager, and the analysis is insufficient to do so. Additionally, we investigate if there are differences between model formulations in estimation of parameter changes, and if the pressure waveform applied as a substitute for aortic hemodynamic measurements is important in this regard.

## Results

### Comparison of estimated parameter changes

**Fig. 1:**
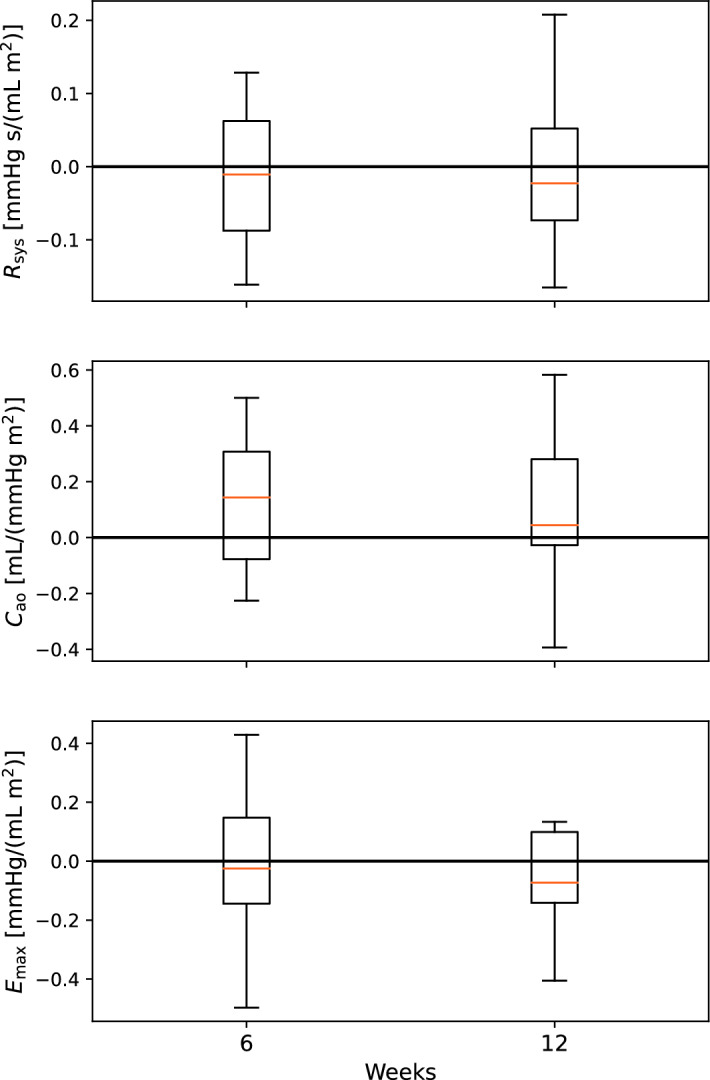
The figure presents the quartiles with whiskers for the changes in parameters relative to the parameters estimated at baseline, or on week 0. These results are made using the carotid pressure waveform and the open-loop model

**Fig. 2:**
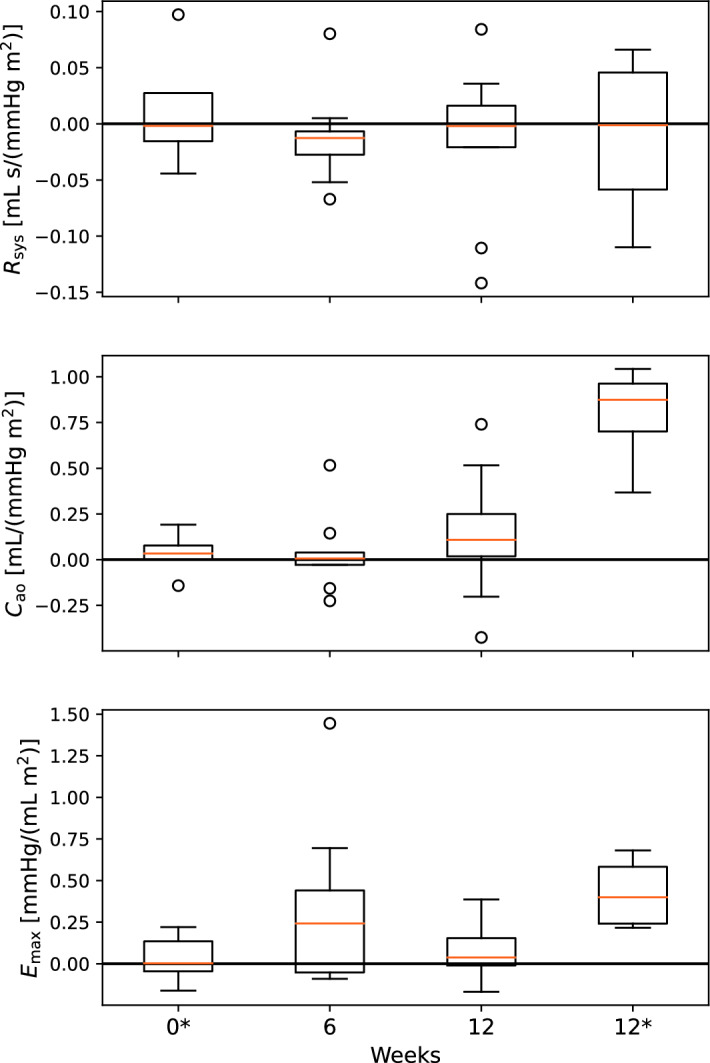
The figure presents the quartiles with whiskers the changes in parameters relative to the parameters estimated at baseline, or on week 0. These results are made using the finger pressure waveform and the open-loop model. The asterisk * indicates that the change is for parameters made 24 h post-CPET for the indicated week number

For ease of readability, most of the closed-loop model results are presented in Appendix B. We calculated the parameter changes between all measurement days. Changes for parameters estimated using either carotid or finger pressure waveforms were then compared to the conventional estimates computed by equations (2–4). Tables 1 and 16 show summary statistics for the parameter changes between any of the three measurement days. The mean change is computed over all participants and for changes between any three measurement days. The mean absolute changes computed by conventional estimates are larger than for the model estimates except for $C_{\text{ao}}$ regardless of model or data, and for $E_{\text{max}}$ for the open-loop model using finger pressure waveforms. $C_{\text{ao}}$ exhibits the largest difference between means by a factor of approximately 2. The parameter changes for the open-loop models are also plotted in Figs. 1 and 2, to illustrate the development throughout the study period. In this case, all changes are computed relative to the initial estimate on measurement day 1. In the appendix, the closed-loop results are presented in Figs. 8 and 9.

**Table 1:** Maximal, minimal, average and average absolute changes for total peripheral resistance ($R_{\text{sys}}$), systemic arterial compliance ($C_{\text{ao}}$), and maximal left-ventricular elastance ($E_{\text{max}}$)

Parameter	Wave-form	Estimation method	Maximal abs. change	Minimal abs. change	Maximal change	Minimal change	Mean change	Mean abs. change	Units
$R_{\text{sys}}$	C	Model	0.208	0.001	0.208	− 0.165	− 0.008(0.090)	0.074(0.052)	$\frac{\mathrm {mmHg \ s}}{\text{mL}\mathrm {m^2}}$
$C_{\text{ao}}$	C	Model	0.583	0.002	0.583	− 0.518	0.058(0.256)	0.206(0.162)	$\frac{\text{mL}}{\text{mmHg}\mathrm {m^2}}$
$E_{\text{max}}$	C	Model	0.497	0.006	0.429	− 0.497	− 0.043(0.215)	0.177(0.128)	$\frac{\text{mmHg}}{\text{mL}\mathrm {m^2}}$
$R_{\text{sys}}$	C	Conv.	0.213	0.000	0.213	− 0.203	− 0.011(0.062)	0.091(0.064)	$\frac{\mathrm {mmHg \ s}}{\text{mL}\mathrm {m^2}}$
$C_{\text{ao}}$	C	Conv.	0.324	0.001	0.265	− 0.324	0.016(0.145)	0.122(0.080)	$\frac{\text{mL}}{\text{mmHg}\mathrm {m^2}}$
$E_{\text{max}}$	C	Conv.	0.793	0.004	0.504	− 0.793	-0.074(0.246)	0.188(0.176)	$\frac{\text{mmHg}}{\text{mL}\mathrm {m^2}}$
$R_{\text{sys}}$	F	Model	0.151	0.002	0.151	− 0.142	-0.011(0.062)	0.045(0.043)	$\frac{\mathrm {mmHg \ s}}{\text{mL}\mathrm {m^2}}$
$C_{\text{ao}}$	F	Model	0.740	0.000	0.740	− 0.425	0.094(0.267)	0.192(0.208)	$\frac{\text{mL}}{\text{mmHg}\mathrm {m^2}}$
$E_{\text{max}}$	F	Model	1.444	0.006	1.444	− 1.058	0.043(0.430)	0.270(0.337)	$\frac{\text{mmHg}}{\text{mL}\mathrm {m^2}}$
$R_{\text{sys}}$	F	Conv.	0.163	0.002	0.157	− 0.163	− 0.019(0.072)	0.055(0.051)	$\frac{\mathrm {mmHg \ s}}{\text{mL}\mathrm {m^2}}$
$C_{\text{ao}}$	F	Conv.	0.243	0.001	0.243	− 0.113	0.065(0.102)	0.098(0.070)	$\frac{\text{mL}}{\text{mmHg}\mathrm {m^2}}$
$E_{\text{max}}$	F	Conv.	0.793	0.019	0.161	− 0.793	− 0.175(0.211)	0.207(0.179)	$\frac{\text{mmHg}}{\text{mL}\mathrm {m^2}}$

Results are given for parameter changes produced by open-loop model optimization and by computation using conventional techniques, based on both carotid (C) and finger (F) pressure waveforms

Maximal and minimal changes are given unsigned, and can be any changes between the first, second and third measurement days

The carotid measurements describe 14 participants with carotid pressure measurements, while the finger pressure includes 9 participants with synchronized flow and pressure

Tables 2 and 17 show the Pearson correlation (*r*) between parameter changes estimated by model parameter optimization, and conventional estimates. The tables describe the correlation between changes between different measurement days in parameters $R_{\text{sys}}$, $C_{\text{ao}}$, and $E_{\text{max}}$ using both choice of pressure waveforms and models. The correlation is between the same changes computed by the CV model optimization versus the standard estimation equations Eq. (2–4). Changes in $R_{\text{sys}}$ are highly correlated in all scenarios. $C_{\text{ao}}$ is mainly moderately to highly correlated for carotid pressure, but we cannot find significant correlation when using finger pressure. $E_{\text{max}}$ has no significant correlation except for moderate negative correlation for carotid pressure for the change from measurement day 1 to 3, and for all changes collected when using the closed-loop model.

**Table 2:** Correlation statistics for parameter changes from total peripheral resistance ($R_{\text{sys}}$), systemic arterial compliance ($C_{\text{ao}}$), and maximal left-ventricular elastance ($E_{\text{max}}$)

Parameter	Wave-form	Temporal change (meas. days)	*r*	*p* value	CI95%
$R_{\text{sys}}$	C	1–2	0.992	$<10^{-3}$	[0.97, 1.00]
$R_{\text{sys}}$	C	1–3	0.988	$<10^{-3}$	[0.96, 1.00]
$R_{\text{sys}}$	C	2–3	0.973	$<10^{-3}$	[0.91, 0.99]
$R_{\text{sys}}$	C	All	0.986	$<10^{-3}$	[0.97,0.99]
$C_{\text{ao}}$	C	1–2	0.649	0.012	[0.18, 0.88]
$C_{\text{ao}}$	C	1–3	0.672	0.009	[0.22, 0.89]
$C_{\text{ao}}$	C	2–3	0.496	0.071	[− 0.05, 0.81]
$C_{\text{ao}}$	C	All	0.618	$<10^{-3}$	[0.39, 0.78]
$E_{\text{max}}$	C	1–2	− 0.385	0.194	[− 0.77, 0.21]
$E_{\text{max}}$	C	1–3	− 0.579	0.038	[− 0.86, -0.04]
$E_{\text{max}}$	C	2-3	− 0.012	0.967	[-0.54, 0.52]
$E_{\text{max}}$	C	All	− 0.267	0.096	[− 0.53, 0.05]
$R_{\text{sys}}$	F	1–2	0.951	$<10^{-3}$	[0.78, 0.99]
$R_{\text{sys}}$	F	1–3	0.990	$<10^{-3}$	[0.96, 1.00]
$R_{\text{sys}}$	F	2–3	0.992	$<10^{-3}$	[0.96, 1.00]
$R_{\text{sys}}$	F	All	0.984	$<10^{-3}$	[0.96, 0.99]
$C_{\text{ao}}$	F	1–2	− 0.105	0.789	[− 0.72, 0.60]
$C_{\text{ao}}$	F	1–3	0.233	0.547	[− 0.51, 0.78]
$C_{\text{ao}}$	F	2–3	0.369	0.329	[− 0.39, 0.83]
$C_{\text{ao}}$	F	All	0.181	0.365	[− 0.21, 0.53]
$E_{\text{max}}$	F	1–2	− 0.206	0.625	[− 0.80, 0.58]
$E_{\text{max}}$	F	1–3	− 0.126	0.767	[− 0.76, 0.64]
$E_{\text{max}}$	F	2–3	− 0.466	0.206	[− 0.86, 0.29]
$E_{\text{max}}$	F	All	− 0.356	0.081	[− 0.66, 0.05]

Results are given for correlations between parameter changes produced by open-loop model optimization and by computation using conventional equations, based on both carotid (C) and finger (F) pressure waveforms

**Fig. 3:**
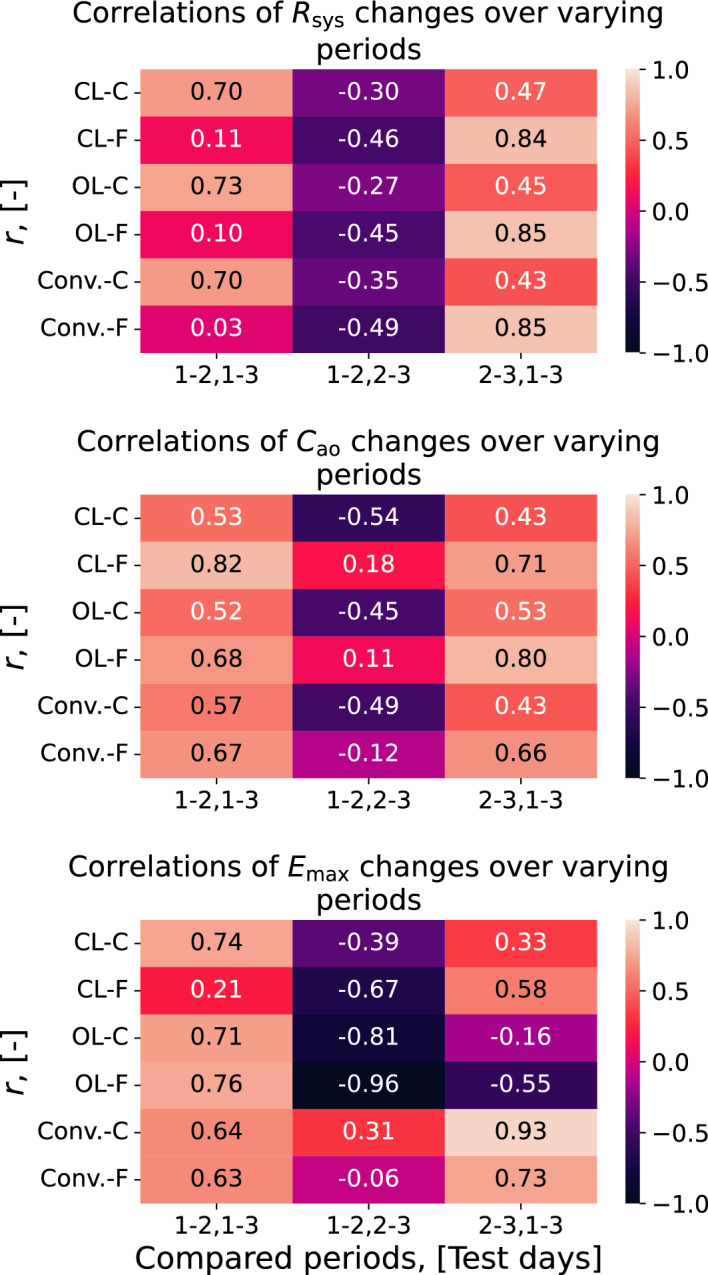
Correlations between parameter changes over the first half, second half and the entire study period for the closed-loop (CL) and open-loop (OL) models. “F” indicates finger pressure waveform, while “C” indicates carotid waveform. From top to bottom panel: $R_{\text{sys}}$, $C_{\text{ao}}$, $E_{\text{max}}$. The “Conventional” (Conv.) estimate correlations are based on the the data with the carotid waveform. The Conv. estimates of $R_{\text{sys}}$ are slightly different for each of the waveforms as the mean pressure is estimated from the calibrated waveform

Figure 3 shows examples of the correlations between parameter changes of different parts for the study period and compares results for both CV models, as well as the different choice of pressure waveforms. Firstly, the correlations for the closed- and open-loop models are very similar in most cases. Secondly, we observe that the correlations are mainly consistent between model estimates and conventional estimates by Eqs. (3, 4), except for $E_{\text{max}}$ where the equation-based conventional estimates often exhibit the opposite behavior to the model estimates. There is also a pattern revealing that changes over the first half of the study period and over second half of the exercise period are often correlated to a low or moderate degree and with a negative sign.

### Longitudinal and post-exercise parameter variability

To assess whether changes monitored after 12 weeks were different to the day-to-day variability and short-term transient exercise effects of the hemodynamics, we computed the parameter changes between measurements made before the CPET and the day after. This was only possible for a subset of the finger pressure waveforms, since only these signals were monitored before and after the CPET. The average parameter changes are presented in Tables 3 and 18. The average change from measurements made before the CPET to measurements made after the CPET are typically on the same order of magnitude as the change after 12 weeks. There are some notable exceptions especially for conventional parameter estimates for left-ventricular elastance and arterial compliance where changes over 12 weeks are two to four times larger than the average changes computed directly before and after the CPET test. Comparing Tables 1 and 16, we see that the range of computed CPET changes are similar to the changes that are computed over longer periods and over all participants. Furthermore, according to this comparison, the average short-term changes connected to a single CPET are very similar to the average change for any of the longer term changes for most parameters. The longer term changes for participants are usually higher for $C_{\text{ao}}$. It should also be noted that for the changes following a single CPET session compared to the change over all 12 weeks, the sign changes for $R_{\text{sys}}$.

**Table 3:** Average and average absolute parameter changes over different periods throughout the study period, describing the parameter change from measurements made before a CPET and the day after

Parameter	Estimation change	Temporal change	Mean abs. change	Mean change
$R_{\text{sys}}$	Model	CPET week 0	0.043(0.040)	0.020(0.060)
$R_{\text{sys}}$	Model	CPET week 12	0.059(0.022)	0.030(0.060)
$R_{\text{sys}}$	Model	Pre-CPET week 0-12	0.050(0.061)	− 0.042(0.069)
$R_{\text{sys}}$	Conv.	CPET week 0	0.052(0.043)	0.016(0.072)
$R_{\text{sys}}$	Conv.	CPET week 12	0.064(0.022)	0.035(0.066)
$R_{\text{sys}}$	Conv.	Pre-CPET week 0-12	0.055(0.066)	− 0.053(0.068)
$C_{\text{ao}}$	Model	CPET week 0	0.076(0.083)	0.076(0.083)
$C_{\text{ao}}$	Model	CPET week 12	0.254(0.349)	− 0.190(0.400)
$C_{\text{ao}}$	Model	Pre-CPET week 0-12	0.327(0.359)	0.327(0.359)
$C_{\text{ao}}$	Conv.	CPET week 0	0.072(0.071)	0.068(0.076)
$C_{\text{ao}}$	Conv.	CPET week 12	0.031(0.024)	− 0.003(0.044)
$C_{\text{ao}}$	Conv.	Pre-CPET week 0-12	0.127(0.069)	0.127(0.069)
$E_{\text{max}}$	Model	CPET week 0	0.141(0.073)	0.037(0.173)
$E_{\text{max}}$	Model	CPET week 12	0.254(0.217)	− 0.030(0.364)
$E_{\text{max}}$	Model	Pre-CPET week 0-12	0.102(0.081)	0.097(0.90)
$E_{\text{max}}$	Conv.	CPET week 0	0.082(0.090)	− 0.082(0.090)
$E_{\text{max}}$	Conv.	CPET week 12	0.070(0.040)	0.070(0.040)
$E_{\text{max}}$	Conv.	Pre-CPET week 0-12	0.281(0.078)	− 0.281(0.078)

Pre-CPET denotes parameter changes calculated between the first and final measurement day based on measurements made prior to the CPET

Results are given for parameter changes produced by open-loop model optimization and by computation using conventional techniques, based on the finger pressure waveforms

### Case study

Based on the observation that the parameter changes based on finger pressure lack clear correlation to the conventional estimate changes in many instances, we drop these in the case and regression analyses onwards. Four participants who fulfilled the selection criteria as outlined in the "Data collection" section have their parameters throughout the study period shown in Figs. 6 and 7 in Appendix C. The plots also show the pairwise relationships between the variables incorporated in the regression analysis.

### Regression analysis

The results for the first two regression analyses to investigate regression to the mean are shown in Tables 4 and 19. These results indicate that the final parameter values are generally moderately to highly positively correlated with the difference between personal and population averages. The resistance and ventricular elastance parameters on the final parameter day are moderately to highly correlated for either model, while compliance is approximately moderately correlated. For the correlation of the parameter change to the difference between the initial parameter value and the population mean, the trend shows that regression coefficients are negative, and with apparently low-to-moderate values of correlation. The adjusted $r^2$ is highest in $R_{\text{sys}}$ and this would also suggest a higher level of regression to the mean compared to the other parameters.

**Table 4:** Ordinary linear regression models for changed model parameters based on the difference between the individual average parameter values over the two first measurement days (subscript: avg) or the baseline parameter value and the population average (subscript: pop)

DV	$R_{\text{sys,3}}$	$C_{\text{ao,3}}$	$E_{\text{max,3}}$	$\Delta _{1,3} R_{\text{sys}}$	$\Delta _{1,3} C_{\text{ao}}$	$\Delta _{1,3} E_{\text{max}}$
Intercept	0.598*	0.969*	0.985*	− 0.012	0.088	− 0.064
$R_{\text {sys,avg}}$-$R_{\text{sys,pop}}$	0.101*					
$C_{\text{ao,avg}}$-$C_{\text{ao,pop}}$		0.166*				
$E_{\text{max,avg}}$-$E_{\text{max,pop}}$			0.307*			
$R_{\text{sys,1}}$-$R_{\text{sys,pop}}$				− 0.056*		
$C_{\text{ao,1}}$-$C_{\text{ao,pop}}$					− 0.111	
$E_{\text{max,1}}$-$E_{\text{max,pop}}$						− 0.045
Adj. $r^2$	0.673	0.337	0.867	0.250	0.129	0.001
N	14	14	14	14	14	14
F-statistic	27.72	7.616	85.62	5.330	2.925	1.014
F-test, p-value	0.000*	0.017*	0.000*	0.040*	0.113	0.334

The asterisk indicates a p-value less than 0.05

The model parameters are optimized for the open-loop model using the carotid pressure waveform

Indices, 1–3 indicate measurement day

DV: dependent variable

For the second part of the regression analysis, we built ordinary and linear mixed regression models for prediction of parameter values or changes based on age, BMI, and gender. We also considered the scatter plots of model parameters versus the measurements to investigate possible variable relationships. The scatter plots are shown in Figs. 6 and 7.

Linear mixed-effects regression models are shown in Tables 5 and 6. In the majority of cases, addition of SV to the baseline model caused the highest level of explained variance, as indicated by the unexplained variance measure (residual variance). Adding solely CRF (C) to the baseline model (A) did not improve the level of explained variance when compared to any of the models incorporating SV (B and D). For resistance, the the various models performed similarly in terms of residual variance, and the coefficients for sex, BMI, SV were consistently significant across both models with sex being the most influential. For arterial compliance only SV was a consistent explanatory factor across models with sex being the most influential. Finally, for ventricular elastance, sex and SV explained the larges amount of variation. While $\text{VO2max}$ was sometimes significant for $C_{\text{ao}}$ in model C the regression coefficient typically changed sign and/or magnitude in model D for all parameters. Patterns in estimated coefficients and unexplained variance are generally similar between both the closed- and open-loop model. An exception of note is that the models for ventricular elastance displays a higher group variance and higher values for the significant coefficients for the open-loop derived parameters.

**Table 5:** Linear mixed-effect regression models of the model parameters total peripheral resistance, systemic arterial compliance, and maximal left-ventricular elastance using the closed-loop CV model

	A	B	C	D
$$R_{\text{sys}} :$$				
Intercept	0.655*	0.635*	0.626*	0.649*
Age	− 0.016	− 0.011	− 0.022	− 0.005
Sex	− 0.143*	− 0.097*	− 0.077	− 0.129*
BMI	− 0.098*	− 0.093*	− 0.118*	− 0.085*
SV		− 0.076*		− 0.069*
$\text{VO2max}$			-0.035	0.023
Unex. Var.	0.005	0.002	0.006	0.003
N	42	42	28	28
Groups	14	14	14	14
Group size	3	3	2	2
Group variance	0.002	0.003	0.002	0.002
$C_{\text{ao}} :$	A	B	C	D
Intercept	0.973*	1.021*	1.127*	1.055*
Age	0.016	0.006	0.103	0.047
Sex	− 0.013	− 0.124*	− 0.424*	− 0.237
BMI	0.054	0.048	0.234*	0.120
SV		0.187*		0.218*
$\text{VO2max}$			0.306*	0.107
Unex. Var.	0.035	0.023	0.028	0.008
N	42	42	28	28
Groups	14	14	14	14
Group size	3	3	2	2
Group variance	0.033	0.005	0.030	0.017
$E_{\text{max}} :$	A	B	C	D
Intercept	0.955*	0.977*	0.930*	0.905*
Age	0.021	0.015	0.004	− 0.018
Sex	0.173*	0.121	0.178	0.246*
BMI	− 0.064	− 0.074*	-0.074	− 0.124*
SV		0.087*		0.102*
$\text{VO2max}$			− 0.014	− 0.092
Unex. Var.	0.014	0.013	0.015	0.016
N	42	42	28	28
Groups	14	14	14	14
Group size	3	3	2	2
Group variance	0.019	0.009	0.022	0.008

The model parameters are estimated for closed-loop model using carotid pressure waveform data. “Unex. Var.” is short for unexplained variance, while N is the number of observations

The regression coefficients are normalized. Asterisks indicate significant coefficients with a p-value less than 0.05

The letters $\mathrm {A-D}$ are labels for the different models

**Table 6:** Linear mixed regression models of the model parameters total peripheral resistance, systemic arterial compliance, and maximal left-ventricular elastance uisng the open-loop CV model

	A	B	C	D
$$R_{\text{sys}} :$$				
Intercept	0.660*	0.643*	0.640*	0.656*
Age	− 0.010	− 0.006	− 0.011	0.003
Sex	− 0.134*	− 0.094*	− 0.089	− 0.134*
BMI	− 0.096*	− 0.092*	− 0.110*	− 0.081*
SV		− 0.067*		− 0.061*
$\text{VO2max}$			-0.020	0.030
Unex. Var.	0.004	0.002	0.005	0.003
N	42	42	28	28
Groups	14	14	14	14
Group size	3	3	2	2
Group variance	0.002	0.003	0.001	0.002
$C_{\text{ao}} :$	A	B	C	D
Intercept	0.937*	0.988*	1.065*	0.985*
Age	0.014	0.003	0.074	0.013
Sex	0.045	− 0.074	− 0.313	− 0.107
BMI	0.069	0.062	0.225*	0.103
SV		0.199*		0.224*
$\text{VO2max}$			0.243	0.028
Unex. Var.	0.034	0.022	0.036	0.013
N	42	42	28	28
Groups	14	14	14	14
Group size	3	3	2	2
Group variance	0.043	0.009	0.033	0.019
$E_{\text{max}} :$	A	B	C	D
Intercept	0.835*	0.890*	0.947*	0.873*
Age	0.036	0.023	0.057	− 0.002
Sex	0.443*	0.315*	0.167	0.359*
BMI	− 0.012	− 0.032	0.096	− 0.034
SV		0.215*		0.214*
$\text{VO2max}$			0.168	-0.033
Unex. Var.	0.024	0.010	0.015	0.011
N	42	42	28	28
Groups	14	14	14	14
Group size	3	3	2	2
Group variance	0.056	0.010	0.080	0.014

The model parameters are estimated for open-loop model using carotid pressure waveform data

“Unex. Var.” is short for unexplained variance, while N is the number of observations

The regression coefficients are normalized

Asterisks indicate significant coefficients with a p-value less than 0.05

The letters $\mathrm {A-D}$ are labels for the different models

The ordinary linear regression models for parameter changes shown in Tables 7 and 8 indicate that trends are similar in terms of patterns of the adjusted $r^2$ across CV models. Further, SV is the most prominent explanatory variable, and model B typically has the highest amount of explained variance. An exception of note is that the change in ventricular elastance has a higher degree of explained variance for the open-loop compared to the closed-loop formulation.

For $R_{\text{sys}}$, increases in CRF and SV correlated with negative change in ($\Delta _{1,3} R_{\text{sys}}$), while increased BMI exhibited the opposite pattern. Change in arterial compliance increases with increased SV, and so does ventricular elastance in both CV model formulations. Change in maximal ventricular elastance is explained by increases in SV, but also by age for the open-loop model, which is not shown to affect this parameter when compared to the linear mixed-effects models.

**Table 7:** Ordinary linear regression models of the model parameters for total peripheral resistance, systemic arterial compliance, and maximal left-ventricular elastance

	A	B	C	D
$$\Delta _{1,3} R_{\text{sys}} :$$				
Intercept	− 0.001	− 0.004	0.004	0.005
Age	− 0.058	− 0.021	− 0.053	− 0.020
Sex	− 0.028	− 0.020	− 0.039	− 0.020
$\Delta _{1,3}$BMI	0.009	0.006	− 0.004	0.006
$\Delta _{1,3}$SV		− 0.081*		− 0.082*
$\Delta _{1,3} \text{VO2max}$			− 0.035	0.013
Adj. $r^2$	0.073	0.615	0.100	0.567
N	14	14	14	14
F-statistic	1.343	6.192	1.361	4.405
F-test, p-value	0.315	0.011*	0.321	0.032*
$\Delta _{1,3} C_{\text{ao}} :$	A	B	C	D
Intercept	0.053	0.062	0.038	0.060
Age	0.082	− 0.024	0.066	− 0.023
Sex	− 0.008	− 0.013	0.044	− 0.008
$\Delta _{1,3}$BMI	-0.004	-0.007	0.038	0.012
$\Delta _{1,3}$SV		0.225*		0.218*
$\Delta _{1,3} \text{VO2max}$			0.111	0.015
Adj. $r^2$	− 0.157	0.683	− 0.027	0.647
N	14	14	14	14
F-statistic	0.414	8.997	0.9156	5.775
F-test, p-value	0.747	0.005*	0.495	0.015*
$\Delta _{1,3} E_{\text{max}} :$	A	B	C	D
Intercept	− 0.103	− 0.102	− 0.099	− 0.096
Age	0.026	0.020	0.030	0.017
Sex	0.143	0.142	0.136	0.128
$\Delta _{1,3}$BMI	0.042	0.043	0.033	0.030
$\Delta _{1,3}$SV		0.015		0.033
$\Delta _{1,3} \text{VO2max}$			− 0.024	− 0.038
Adj. $r^2$	0.189	0.108	0.124	0.055
N	14	14	14	14
F-statistic	2.009	1.393	1.461	1.153
F-test, p-value	0.177	0.311	0.292	0.408

The model parameters are estimated for closed-loop model using carotid pressure waveform data. “Adj. $r^2$” is the adjusted $r^2$, while N is the number of observations

The regression coefficients are normalized

Asterisks indicate significant coefficients with a p-value less than 0.05

The letters $\mathrm {A-D}$ are labels for the different models

**Table 8:** Ordinary linear regression models of the model parameters for total peripheral resistance, systemic arterial compliance, and maximal left-ventricular elastance

	A	B	C	D
$$\Delta _{1,3} R_{\text{sys}} :$$				
Intercept	0.002	− 0.001	0.006	− 0.002
Age	− 0.053	− 0.018	− 0.049	− 0.017
Sex	− 0.032	− 0.026	− 0.042	− 0.024
$\Delta _{1,3}$BMI	0.012	0.008	0.001	0.010
$\Delta _{1,3}$SV		− 0.075*		− 0.078*
$\Delta _{1,3} \text{VO2max}$			− 0.029	− 0.006
Adj. $r^2$	0.061	0.588	0.055	0.540
N	14	14	14	14
F-statistic	1.280	5.638	1.189	4.058
F-test p-value	0.334	0.015*	0.379	0.039*
$\Delta _{1,3} C_{\text{ao}} :$	A	B	C	D
Intercept	0.069	0.077	0.061	0.082
Age	0.128	0.040	0.112	0.038
Sex	0.043	0.025	0.062	0.014
$\Delta _{1,3}$BMI	− 0.021	− 0.012	0.000	− 0.023
$\Delta _{1,3}$SV		0.187*		0.202*
$\Delta _{1,3} \text{VO2max}$			0.058	− 0.031
Adj. $r^2$	0.020	0.548	− 0.022	0.509
N	14	14	14	14
F-statistic	1.090	4.943	0.931	3.694
F-test p-value	0.398	0.022*	0.448	< 0.050*
$\Delta _{1,3} E_{\text{max}} :$	A	B	C	D
Intercept	− 0.049	− 0.045	− 0.053	− 0.041
Age	0.139*	0.089*	0.135*	0.087*
Sex	− 0.035	− 0.045	− 0.026	− 0.054
$\Delta _{1,3}$BMI	− 0.052	− 0.047*	− 0.042	− 0.056*
$\Delta _{1,3}$SV		0.106*		0.118*
$\Delta _{1,3} \text{VO2max}$			0.026	− 0.026
Adj. $r^2$	0.432	0.856	0.402	0.867
N	14	14	14	14
F-statistic	4.294	20.38	3.182	23.16
F-test, p-value	0.034*	0.000*	0.069	0.000*

The model parameters are estimated for open-loop model using carotid pressure waveform data. “Adj. $r^2$” is the adjusted $r^2$, while N is the number of observations

The regression coefficients are normalized

Asterisks indicate significant coefficients with a p-value less than 0.05

The letters $\mathrm {A-D}$ are labels for the different models

## Discussion

Through a combination of correlation, regression and case analysis we identified that the parameter changes computed by CV model optimization do reflect some of the patterns found by more traditional methods, but also that the results exhibit patterns consistent with regression to the mean. Further, we found no patterns which indicated that change in cardiorespiratory fitness informed the changes. In the interest of tracking changes in hypertension management, these results are unsatisfactory in answering whether the model and estimation approach are suitable for the purpose. While the arterial parameters were relatively consistent with other estimation methods, the ventricular elastance did not exhibit similar behavior. However, further pursuit of answering if such a personalization framework can function with a limited data set, with a model capable of simulating hemodynamic changes and stimuli for giving insight into the individual’s response, may still produce a valuable tool in the future of personalized medicine.

Total peripheral resistance has been reported to decrease after bouts of physical activity, both in the short and long term [11, 18]. The size of this decrease is also dependent upon the baseline blood pressure, type of physical activity, and the individual's properties. As a consequence, the trend of marginally lowered systemic resistance seen in these results on average (see Tables 1, 3, 16, and 18) is in agreement with previous findings. A trend of decreased resistance is also observed in several of the participants chosen for the case analysis.

By comparing the computed changes between measurement days and comparing to the changes pre- to post-CPET, there did not seem to be any clear signal of sustained exercise-induced remodeling in parameters over the 12 weeks which was consistently different than the transient short-term post-CPET effects. This may have been due to insufficient data, insufficient physical activity or lack of response to exercise in these individuals, as only one of the four participants with CPET measurements met the criteria for and is included in the case study. A criteria that was determined by who saw the largest change in VO2max after the exercise intervention. One cannot rule out that there are possible non-responders to exercise in this study sample either.

Comparing the correlation of parameter changes between different parts of the study period (Fig. 3), we note that the changes in the first half of the study period usually tend towards a negative and low correlation with the changes of the second half. Although these correlations are not necessarily significant, this is normally recognized as a pattern which may indicate that the parameter values regress to a personal mean. This would mean that the changes may be extreme observations by chance, and are not necessarily caused by the study intervention. Additionally, the correlation patterns are more similar between parameters changes estimated using the same pressure waveforms and CV model, than when using the same CV model with different pressure waveforms. This suggests that the pattern of changes are more dependent upon measurement modality than the CV models themselves. Furthermore, if there actually is statistically significant regression to the mean, it would also be a sign of no or little effect of exercise on the parameter changes. The CV model parameter changes for resistance and compliance seem to correlate reasonably well with the data from conventional estimates (see Tables 2 and 17). This would suggest that changes in parameter estimates made for the CV models are informed by changes in the data and are not purely results of uncertainty or poor performance by the estimation method. Even though the change in hemodynamics may not be convincingly informed by exercise, the fact that the pattern of changes in many cases agree based on different estimation approaches supports that both methods produce similar week-to-week variations as expressed by the data. Ventricular elastance on the other hand does not show the same behavior in all cases, and often has a negative relationship. The results also indicate that the carotid pressure waveform more often produces estimates higher correlated with the conventional parameter change estimates. The correlation patterns identified here are similar for both CV model formulations.

For the closed-loop model and case analysis, we found the following: total peripheral resistance decreased compared to baseline in two out of four participants. The arterial compliance parameter increased in four out of four participants. Maximal ventricular elastance increased in one out of four participants. Only estimates for one participant saw all of the listed changes simultaneously, which indicates that these parameters saw changes expected to lower blood pressure or improved cardiac function. For the open-loop model, the case analysis gives the following observation: total peripheral resistance decreased compared to baseline in two out of four participants, while maximal ventricular elastance increased. Arterial compliance increased in four out of four participants. The parameters in two participants saw all of the listed changes simultaneously. Hence, the open-loop model seems to express a pattern of consistent remodeling in more participants than the closed-loop model, although this is very limited data and possibly only marginal differences.

The regression analysis was subject to scarce data, and regression coefficients were rarely significant, such that these results cannot not reliably prove any influence and the trial study was not designed for this. However, the models may instead give an indication of whether physical activity or fitness did influence parameter estimates and if trends support what is expected from the literature. Therefore, we also attempt to interpret trends even for non-significant coefficients.

In Tables 4 and 19 the difference of the personal mean and population mean exhibit medium to high adjusted $r^2$ for most parameters on the final measurement day. In other words, the more extreme the final value is, the more extreme the personal average is likely to be. For the relationship between parameter change and the population mean subtracted from the initial parameter value, low-to-medium values of adjusted $r^2$ were observed. For the closed-loop model the arterial compliance exhibited the highest $r^2$ value, but the peripheral resistance was similarly highest in the open-loop model. These two parameters could therefore be more likely to be expected to regress to the population mean for their respective models than the other parameters. As observed in 3, a pattern of regression to a personal mean is supported by this analysis. Combined, these two observations suggest that some of the changes are caused by chance. These results and the observed correlation between estimation methods could both at least partially be explained by day-to-day variability in individuals fluctuating about a personal mean.

The linear mixed-effects models indicate that for $R_{\text{sys}}$ practically all covariates have a negative coefficient. This negative relationship is consistent with prior studies and physiological understanding of how improved fitness and vascular remodeling result in increased cardiac output through improved conduit function of the vasculature. They would be expected to be negative as increased SV, BMI and $\text{VO2max}$ is expected to lower the resistance value due to, for example, increased cardiac output, and improved fitness. For $C_{\text{ao}}$ there are mainly positive coefficients with the exception of sex. This is in agreement with the expected effect of increased SV (while maintaining blood pressure) and $\text{VO2max}$. For increased age and BMI, vessels are expected to stiffen, but the age range of included participants may be insufficient to detect this. Finally, for $E_{\text{max}}$, we find negative coefficients for BMI and $\text{VO2max}$. A full understanding of the relationship between BMI and ventricular contractility has not been established from prior works. Manoliu et al. estimated end-systolic elastance and found that contractility slowly increased with BMI in middle-aged subjects [19], but other studies suggest that obesity decreases with other load-independent contractility indices in people with hypertension [20]. Fernandes-Silva et al. observed increased end-systolic elastance with increased BMI in the elderly also when adjusting for age [21]. Similarly, some findings have indicated contractility to increase in terms of EF after exercise [11], so we would expect to find positive coefficients for $\text{VO2max}$ if increased fitness has an effect. While different notions for cardiac contractility are used to describe human hearts and can be contradictory, Oláh et al. observed increased resting end-systolic elastance in rats after 12 weeks of exercise [14]. Positive coefficients for SV seem reasonable as increased contractility could lead to a higher SV by ejecting more blood per heart beat by, for example, an increased EF.

The ordinary linear regression analysis of parameter changes as shown in Tables 7 and 8, give additional perspectives. Increases in SV and $\text{VO2max}$ tend to correspond with decreased $\Delta _{1,3} R_{\text{sys}}$. Conversely, resistance decreases when the change in SV and $\text{VO2max}$ is positive, which is what we expected physiologically. On the other hand, the change of resistance seems to weakly increase with BMI. Change in arterial compliance increases with increased SV, and so does ventricular elastance in both CV model formulations. Ventricular elastance change is explained mainly by SV, and age, but it is also an exception in that it has a significantly higher level of explained variance for the open-loop model parameters compared to the closed-loop formulation parameters.

Taking the approximated levels of measurement uncertainty into consideration from the "Measurement uncertainty" section, we find that for the OLR analysis the best models, which include SV, leave in the neighborhood of 40% unexplained variance. Seeing as the highest level of measurement uncertainty is also introduced by SV, it is possible that a part of this unexplained variance comes from this uncertainty. The remaining variance could possibly be explained by the variability in some of the other model parameters, some unknown explanatory variables that were not collected or in systematic measurement errors we have failed to take into consideration. For the linear mixed-effects models, the variance seem to be explained to a higher degree by the different individuals, than by lacking measurement error.

It should also be noted that a weak signal for remodeling may be caused by low activity levels as only eight out of initially 26 participants increased their average weekly PAI from less than 50 PAI to over 100 PAI over the course of the study period. Previous studies have shown that reaching a 100 PAI weekly reduces risk of CV disease and extends lifespan as compared to those who do not reach this target [15, 16]. We would therefore expect the individuals who achieved this to be more likely to experience remodeling. However, $\text{VO2max}$ is the more well established measure in the context of improved fitness, and by extension cardiorespiratory remodeling, and was therefore preferred in the case analysis. All parameters for both CV models seem to be best explained by the addition of SV as an explanatory variable, and therefore suggests that the model parameters capture the hemodynamic changes from day to day or week to week, as opposed to any influence of physical activity in this study.

Studies by Audebert and Gerringer et al. use lumped-parameter models constructed in similar ways to the one used in this work to track vascular parameters in rats [7, 8]. The former of the two studies, examines the hepatic vessel’s vascular resistances after pharmacologically induced liver cirrhosis and their role in altering the hemodynamics. The paper demonstrates a mean of differences in hepatic arterial resistances of $-$38.0% compared to control rats after 12 weeks of cirrhosis development in a small sample of rats. After 18 weeks, the change is much more prominent; however, at $-$85.8%. Gerringer et al. induced pulmonary hypertension in 27 rats with 17 control animals over a period of 4 weeks and investigated vascular parameter differences in 3- and 4-element Windkessel models at different disease stages. After 4 weeks, significant changes in the hypertensive group was found in both pulmonary resistance (+185.7%) and compliance ($-$69.8%). Similarly between 2 and 4 weeks significant differences were found to be 105.1% for resistance and $-$60.1% for compliance. These results suggest it is possible to distinguish between states of disease and health. However, both studies differs from ours by study subjects, what stimuli is enacted, choice of model and what parameters are included in the model. The mean changes in both studies are, most often, at least an order of magnitude larger than the mean changes observed in this study.

Results from Bjørdalsbakke et al. indicate that the closed-loop model does not have any considerable advantage compared to the open-loop model in terms of resolving parameter changes [17]. Results from the current study suggest that the open-loop model may be better suited for detecting parameter changes. The supporting evidence is that maximal left-ventricular elastance changes can be explained with higher confidence using the open-loop framework. Therefore, analogously to results by Itu et al. [22], an open-loop model with only the adjacent vasculature could be sufficient to determine a selection of highly influential mechanical parameters, describing the pressure–volume loop and global arterial hemodynamics. However, Itu et al. did not explore if this was sufficient to monitor a system in different states of exercise or under CV disease, which remains unknown and is likely problem dependent.

Ultimately, the comparison of the two CV models yields information about whether different model complexities impact what information is reflected by the parameter estimates given the chosen estimation method. The closed-loop formulation is partially described by a stressed blood volume parameter, which the open-loop does not include as its total blood volume can vary during each heart cycle. Total stressed blood volume is highly influential on blood pressure levels in this model [23], but can add complexity to personalization procedures as this parameter affects the state variables of all model compartments and can possibly interact with several other parameters simultaneously. Therefore, we investigated whether the personalization of each model variant by a given personalization procedure captured the same parameter dependencies or whether one of the models was better suited to track parameter changes which were expected to come from, for example exercise stimulus, or other explainable causes. From the initial regression results, it seems that both models yield parameters that may to some degree regress towards a population mean, but mainly $C_{\text{ao}}$. Additionally, all parameters, exhibit some signs that parameters for the individual may regress towards a personal mean (see Fig. 3). And neither model exhibits any convincing evidence that the levels of fitness changes resulting from the clinical trial explains any additional variance in the parameters. But we cannot conclude whether this is due to a low level of exercise remodeling in participants, or whether the models are not able to detect these effects on average. Case analysis shows that parameters for both models can change in a manner expected to be beneficial remodeling after exercise. The models also seem to be able to track changes in parameters similarly to other conventional techniques given variations in the data within and between individuals, but to varying degrees of correlation.

As previously noted, correlations between CV model parameter changes and conventional change estimates are higher for the carotid pressure waveform, suggesting that carotid waveforms may be more useful for computing more realistic and accurate changes (Tables 2 and 17) than the finger pressure waveforms. The changes are on average larger in absolute magnitude for the arterial parameters using the carotid waveform, but the opposite for $E_{\text{max}}$, where the finger pressure waveform estimates result in the larger changes (Tables 1 and 16). We would initially expect this as the waveform is closer to the arterial waveform in shape, but this study allowed us to investigate if the estimated changes using the different waveforms were equally informative or useful. In summary, the changes computed for estimates based on the carotid waveform are more informative than the finger pressure-derived estimates, when compared to conventional estimation methods.

### Limitations

This study is limited by the data set as well as the CV model formulations. The CV models are simplified models of the circulation and do not resolve individual vessels and neglect entire parts of the circulation, such as the pulmonary circulation. Parameters can represent the function of several vessels, and effects such as inertance in larger vessels is neglected. This is a consequence of model simplification, which in our case simplifies parameter estimation at the cost of prediction accuracy and predicted features. The heart model is represented with a periodic elastance model, where the right ventricle and the atria are omitted. For the closed-loop model formulation, the heart chamber acts as both the right and left ventricles as the systemic veins terminate in the mitral valve. Heart valves are modeled as perfect unidirectional diodes which do not allow backflow.

Next, the data foundation is limited. Firstly, in the sense that there are few data points, the statistical analysis is restricted both in robustness and that it cannot account for many interaction terms. Secondly, the exercise trial design was not designed such that all participants would have a uniform amount of exercise. The type of exercise the participants engaged in was not controlled either, apart from monitoring their heart rate, so the exercise response may be variable among participants. The data are either way too sparse to establish a dose–response relationship. Therefore, we have emphasized comparison of the identified patterns to established knowledge, rather than make new conclusions about the effect of exercise based on the data set and models.

## Conclusion

The explanatory analysis shows that the cardiovascular model parameter changes correlate at least moderately well with changes computed from more conventional estimation techniques. This applies for arterial parameters using the carotid pressure waveform. This result suggests that the estimation method and model are able to at least partially capture changes in the data from week to week. The estimates of $E_{\text{max}}$ are not often clearly correlated with conventional estimates, and it is therefore harder to argue that they are sensitive to the changes captured by the hemodynamics. For participants included in the case study, the arterial parameters for over half of participants experienced changes in the direction expected from an increased amount of exercise. However, by analyzing the study population using regression models, we found no clear effect of cardiorespiratory fitness influence on the model parameters representing arterial compliance, resistance and maximal left-ventricular elastance. The model-based mean absolute changes over the study period were not considerably larger or smaller than estimated changes from before to after a CPET test within a span of 24 h. To be able to learn more about parameter changes we recommend focusing on carotid or more central waveforms, as parameters based on these correlate better with conventional estimates than finger pressure-based estimates, despite their relative ease of collection. Additionally, aside from slightly better correlation of $E_{\text{max}}$ estimates between estimation methods, few other results indicate that there is a considerable benefit to using a closed-loop model in terms of tracking parameter changes even though it describes more details of the cardiovascular system. The open-loop model produces estimates for maximal ventricular elastance that yield a higher degree of explained variance in the regression analysis, and thereby detects more explanatory factors for the parameter. Regression models suggest that adding information about $\text{VO2max}$ cannot explain more of the variability in parameter estimates in a majority of cases. This suggests that the remodeling effect is either too small, or the model and parametrization procedure is unable to track the changes reliably. Then the majority of the explanation of the computed parameter changes lies in week-to-week or day-to-day changes, as changes in SV are found to be better at explaining the parameter variability, but uncertainty in the model optimization cannot be ruled out as an explanatory factor.

## Methods

### Study design, setting, and participants

The data originated from a pilot randomized controlled trial for exercise motivation. The study participants were randomly split into two groups. One group was asked to achieve a score of of over a 100 weekly PAI points by using a mobile application reading data from their wrist-worn heart-rate sensor. The second group was instructed to follow the World Health Organization’s general recommendations for physical activity [24]. A total of 26 adults (13 females), 45–65 years of age, met the inclusion criteria at screening: hypertensive (blood pressure $\ge 140/90$) or pre-hypertensive (blood pressure $\ge 140/90$ mmHg), currently physically inactive (self-reported < 50 PAI per week), not using antihypertensive medication and no history of CV disease, secondary hypertension or diabetes. One participant dropped out due to becoming unable to perform exercise for a longer period during the trial period. The the study period lasted for 12 weeks, and hemodynamic measurements were made at the beginning, after 6 weeks and at the end. We refer to these as measurement days 1, 2, and 3. Parts of the trial have previously been described by øyen [25] and Bjørdalsbakke et al. [23]. The characteristics of the study population can be found in Table 9.

By applying a lumped-parameter model of the systemic circulation to the data from the trial, the mechanical properties of the circulation were estimated for specific individuals as described in previous work [17]. We continued on to specifically investigate the left-ventricular contractility by way of maximal left-ventricular elastance ($E_{\text{max}}$), arterial stiffness by arterial compliance ($C_{\text{ao}}$), and arterial vascular resistance by total peripheral resistance ($R_{\text{sys}}$).

The code used in the analysis is available from github.com: https://github.com/nilibjo/NLB_P3_ExaminingTemporalChanges.

**Table 9:** Baseline characteristics of the study population

	*n* = 25
Height	174.3 ± 8.9 cm
Weight	85.9 ± 14.2 kg
BSA	2.0 ± 0.2 m$^2$
BMI	28.2 ± 3.6 kg/m$^2$
Age	55.9 ± 3.9 years
Sex M/F	13/12
SBP	138.5 ± 12.6 mmHg
DBP	87.3 ± 8.7 mmHg
$\text{VO2max}$	36.4 ± 6.8 mL/(kg min)
24h ABPM awake SBP	141.0 ± 13.5 mmHg
24h ABPM awake DBP	85.4 ± 9.1 mmHg
SV (4D)	85.5 ± 20.0 mL
SV LVOT	79.7 ± 20.2 mL

BSA: body surface area; BMI: body mass index; ABPM: ambulatory blood pressure monitoring; SV: stroke volume; LVOT: left-ventricular outflow tract

SBP and DBP signifies systolic and diastolic office blood pressure measurement

4D refers to 3D measurement averaged over time

#### Summary of the clinical trial study protocol

The aim of the clinical trial was to determine how personalized physical activity goals, in terms of PAI, would compare in reducing ambulatory blood pressure levels and improving CV risk profiles compared to participants following recommended national physical activity guidelines. The study was organized as a randomized controlled trial where the intervention group followed physical activity recommendations according to PAI score and the control group followed national guidelines. Participants were invited for screening, where they were asked to fill out a questionnaire, and blood pressure was measured to determine whether they had blood pressure consistent with a normotensive, pre-hypertensive or hypertensive range. 26 participants were found to be eligible for inclusion in the trial. The inclusion criteria was that they had blood pressure measurements in a pre-hypertensive range or above. They were not on any medication for hypertension, had not previously been diagnosed with hypertension, and had no history of diabetes or CV disease. Additionally, their self-reported activity level would amount to less than 50 weekly PAI, meaning that their life styles were quite sedentary and that they had a lower than ideal activity level before participating in the trial.

After this point, the participants were given heart rate monitors to wear for a baseline week, where they were asked to engage in normal life and not make any effort to participate in physical activity outside their norm. The group was split into two groups after this week. Group A (14 participants, 7 male) were asked to aim to participate in 150 min of moderate-intensity exercise per week according to the WHO’s recommendations. These participants were not able to see their PAI score as measured by their heart rate monitors during the trial. Group B (12 participants, 7 male) were advised to gain at least 100 PAI per week, and could monitor their score during the study period.

Upon the end of the baseline week, the intervention period was initiated by equipping the participants with automated Oscar 2 (Suntech) devices for ambulatory blood pressure measurement. The first day of the study period participants were asked to come in to the clinic to have measurements of height, weight, and office blood pressure taken, and to perform a full body scan by using a Inbody 770 bioelectrical impedance system. Subsequently pulse wave velocity was measured. After 10 min of rest, pulse wave velocity and traces of carotid and femoral artery blood pressure waveforms were acquired by applanation tonometry using a Sphygmocor CvMS v9 (AtCor Medical) system as a by-product of recording the carotid–femoral pulse wave velocity. At a different time point, the participant was asked to lie down in left-lateral recumbent position to perform a full transthoracic echocardiographic examination by trained technicians using VingMed Vivid E95 (GE) systems. While maintaining their position, the blood pressure Finometer PRO (Finapres) or NIBP Nano (Finapres) system was mounted on their right hand, and the finger arterial pressure measurements were initiated, while simultaneously making a echocardiographic measurement of the left-ventricular outflow tract blood flow waveform measurement. Synchronization points were made manually by having the participant cough and twitch their finger at a countdown while the operator made a simultaneous annotation in the blood pressure recording system. The third unperturbed cycle prior to the synchronization signal were taken as data cycles. The cycles were subsequently manually matched at diastole in order to get a more precise synchronization. Following the echocardiographic examination the subjects were asked to engage in a cardiorespiratory exercise test (CPET) on a treadmill to measure VO2max. For a subgroup of 8 of the participants, they were also asked to return the following day after 24 h to repeat the echocardiographic examination and finger artery pressure measurements.

**Fig. 4:**
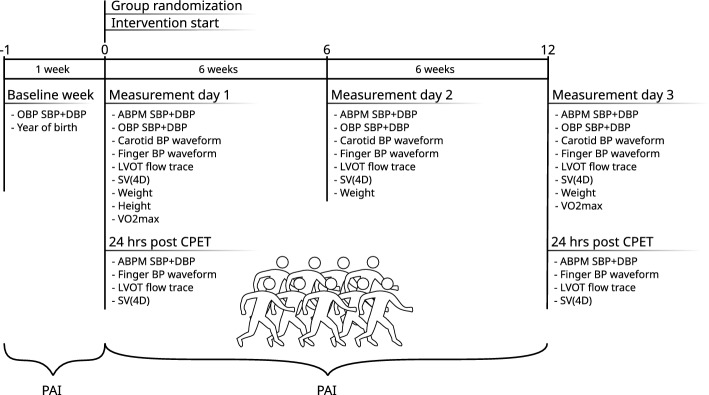
Illustration of the trial timeline with the timing and different types of measurements

Measurements were repeated for all participants after 6 weeks into the study period, and after 12 weeks, i.e., measurement days 2 and 3. The CPET, and repeated echocardiographic measurements were only made after measurement days 1 and 3. The trial timeline is illustrated in Fig. 4.

### Inclusion criteria for analysis

For the analyses within this study, the trial population was further subdivided according to what data were available to each participant, and which analysis techniques they consequently could be included in. Finally, four groups of participants were formed: participants with synchronized finger pressure signals and flow, participants with carotid pressure waveforms paired with flow, participants with finger pressure measurements following a cardiopulmonary exercise test, and participants with the most improvement in $\text{VO2max}$ (the top quartile).

#### Inclusion criteria for correlation and regression analysis

The requirement for inclusion was having blood pressure measurements, LVOT flow and at least one type of pressure waveforms, as well as body mass data for all three measurement days. As a consequence, all drop-outs are excluded. Further, $\text{VO2max}$ estimates should exist for both measurement day 1 and 3. Height data were also required at measurement day 1. For adherence to the exercise intervention, we required that the participants wore their heart-rate monitors for at least 75% of the days of the intervention period.

For blood pressure, awake measurements from 24 h ABPM measurements, or alternatively OBP measurements where ABPM data were missing were required. For blood pressure waveforms, either finger pressure waveforms or carotid pressure waveforms were acceptable. Finger pressure also required synchronization data to LVOT flow in the raw data files. In terms of SV data, 4D SV was preferred, but SV derived from LVOT flows was sufficient if the former was missing.

Due to drop-outs, missing data, or missing synchronization data we selected 55 eligible sets of measurements for synchronized finger pressure and LVOT flow. Among these measurements 9 participants were identified with complete records. Similarly, for the data sets with carotid pressure, 14 eligible participants had complete records. The characteristics for the selected groups using differing pressure waveforms are found in Appendix A.

These inclusion criteria apply for all subsequent analyses as well.

#### Inclusion criteria for analysis of CPET-induced change

The CPET induces changes in hemodynamics, and bouts of exercise have been observed to impact hemodynamics and specifically blood pressure for up to 24 h afterwards [26]. This analysis was made to compare the change observed over longer periods to the change after one bout of strenuous exercise, or to daily variability if exercise effects have attenuated after 24 h. This would indicate whether the model would see any difference or resolve this change similarly.

Inclusion criteria for the sub-analysis on changes after the CPET we require the same blood pressure and echocardiography measurements as described in previous sections on the days immediately following measurement day 1 and 3. By also requiring complete records for flow and finger pressure alignment, this left 4 participants out of the 9 identified in the previous paragraph. Consequently, these 4 participants had complete pressure and flow measurements within 24 h after CPETs conducted at the beginning and end of the intervention (day 1 and 3).

#### Inclusion criteria for individual participant case analysis

In this sub-analysis, we included the participants expected to have exercise-induced CV remodeling based on the measured cardiorespiratory fitness. Individual CV response to physical activity over both the short- and long-term is likely dependent upon properties in the individual which also causes challenges in predicting which type and amount of exercise is sufficient to expect measurable CV remodeling.

To investigate the parameter changes of those we expected to be most likely to experience CV remodeling, we selected participants based on change in $\text{VO2max}$. We set the inclusion criteria to be that the participants should fall in the upper quartile of measured changes in $\text{VO2max}$. This left 4 participants.

### Data collection

Varied hemodynamic data were recorded during the course of the study in the form of blood pressure, aortic flow, and pulse wave velocity. The cardiorespiratory fitness (CRF) was also measured as $\text{VO2max}$ through a cardiopulmonary exercise test (CPET) at measurement days 1 and 3 of the study period. Additionally, the activity levels throughout the study period was monitored using wrist-worn heart rate monitors. Waveform data preprocessing has been described previously by Bjørdalsbakke et al. [17]. The types of measurements are listed in Table 10.

**Table 10:** A summary of measured variables from the clinical trial

Category	Measurements
Activity and fitness	PAI [−], $\text{VO2max}$ [mL/kg/min]
Blood pressure	ABPM SBP+DBP [mmHg]
	OBP SBP+DBP [mmHg]
	Carotid BP waveform trace [−]
	Finger BP waveform [mmHg]
Blood flow	SV [mL], LVOT flow trace [−]
Other	Year of birth [−], height [cm], weight [kg]

ABPM, ambulatory blood pressure measurement; OBP, office blood pressure; SBP, systolic blood pressure; DBP, diastolic blood pressure

#### Physical activity monitoring

Physical activity was monitored in terms of PAI, and the effect on cardiorespiratory fitness was monitored in terms of $\text{VO2max}$.

Physical activity monitoring was made possible by wearable wrist-worn heart rate monitors (LYNK2). The collected heart rate data were aggregated into daily PAI scores representing the physical activity level over the past week. The study group which was asked to achieve a 100 weekly PAI were able to see their PAI scores during the study intervention, while the other group asked to follow the current recommendations were not able to monitor their PAI score.

A cardiopulmonary exercise test (CPET) was conducted for participants on measurement days 1 and 3. The test was performed using a treadmill (Woodway PPS 55) with Metalyzer II (Cortex).

Participants warmed up for 15 min at approximately 70% of estimated maximal heart rate. After warm-up workload was increased by 0.5–1 km/h and/or 1–2% inclination per minute until volitional exhaustion or $\text{VO2max}$ criteria were met. $\text{VO2max}$ was defined as a plateau in $\mathrm {VO_2}$ despite increase in workload and respiratory exchange ratio >1.05. 22 of initially 26 participants reached this requirement, and therefore when $\text{VO2max}$ is referred in this text, we actually mean $\text{VO}_{\text{2,peak}}$ in some cases.

#### Blood pressure recordings

Ambulatory blood pressure monitoring (ABPM) of brachial artery blood pressure over 24 h was measured using Oscar 2 model 250 (SunTech Medical Inc). Conventional office blood pressure (OBP), which is also a brachial artery measurement, was measured using a TangoM2 automatic blood pressure monitor (SunTech Medical Inc). The full pressure waveform was also measured non-invasively in the carotid artery, and finger arteries.

Office blood pressure was recorded in both arms upon screening, and the arm with the highest blood pressure was chosen for all subsequent measurements on all measurement days. Three measurements were taken, with 1.5-min rest between each measurement. Participants were seated throughout the procedure. OBP measurements were taken for all measurements days. For 24-h ABPM, measurements were taken at 30-min intervals at night, and 20-min intervals by day. ABPM was performed for all measurement days.

Digital artery pressure waveforms in the finger were acquired by a Finometer PRO system (FinaPres) for 4 participants, while the remaining participants were measured by a Non-Invasive Blood Pressure Nano system (FinaPres). We refer to this type of measurement as finger pressure in the following. Participants were placed in the left-lateral recumbent position during recordings, and measurements were made in the right hand. Measurements could be made in the index, middle or ring finger depending on where a clear signal was found. The pressure waveforms were subsequently calibrated to brachial blood pressure values obtained by ABPM during participants’ waking hours.

We measured synchronized flow and finger pressure before performing the CPET test at both measurement day 1 and 3. Then the next day, and within 24 h of conducting the CPET, the measurements were repeated for a subset of the participants. On these extra measurement days OBP and 24 h ABPM was also measured. These measurements only exist for finger pressure waveform data, not for the carotid waveform.

A SphygmoCor (CvMS v9, AtCor Medica) system was used to trace the pressure waveform of the carotid artery by applanation tonometry, during the process of estimating the carotid–femoral pulse wave velocity. We also rescaled the resulting waveforms to correspond to ABPM measurements.

#### Echocardiography

A transthoracic echocardiographic examination was performed on all measurement days. In particular, the left-ventricular outflow tract flow (LVOT) flow trace was synchronized to finger pressure recordings during measurement, while the participant was lying in a left-lateral recumbent position. 4D recordings of stroke volume (SV) were also acquired. The Finapres finger cuff was mounted upon the fingers of the participants' right hand while in position.

Taking the traced carotid artery waveforms, we paired them with the same aortic flow waveforms which the finger pressure waveforms were synchronized to.

#### Measurement uncertainty

For blood pressure measurements using ABPM, the devices should satisfy the European Society of Hypertension requirements for accuracy [27]. Consequently, at least 60% of measurements should be within ± 5 mmHg of error compared to measurements made by trained personnel using mercury manometers. Approximating this as the standard deviation of single blood pressure measurement, the average ABPM blood pressure values have a best-case measurement uncertainty standard deviation of ±0.7 mmHg. This approximates a lower bound and is calculated for the arithmetic mean of measurements over a 16-h waking period at 20 min apart using Gauss’ law of error propagation. The approach also assumes the unrealistic case that the measured individual maintains the exact same hemodynamic state throughout the entire period, and no correlation between measurements.

$\text{VO2max}$ measurements were acquired by ergospirometry. According to the manufacturer’s user manual Metalyzer II and Metalyzer 3B has a 2% measurement accuracy in expired gas volume and a 0.1% accuracy in oxygen volume according to the manual. A recent article comparing differences in $\text{VO2}$ measurement errors between different equipment manufacturers by Van Hooren et al. found an average measurement error of 2.85% for Metalyzer 3B [28]. Of course, the process of identifying the maximal value can also introduce errors, but this warrants further analysis beyond the scope of this work.

Kitano et al. have reviewed that for semi-automatic 3D estimation of stroke volume from echocardiography using equipment by different manufacturers [29]. A pooled measurement bias of approximately $-$39.3 mL and $-$19.6 mL was found for semi-automated acquisition of end-diastolic (EDV) and end-systolic (ESV) left-ventricular volumes as compared to measurements by cardiac magnetic resonance imaging (CMRI). SV was not reported directly but ejection fraction could not be shown to be significantly biased. We take CMRI as the ground truth in this case. Suehiro et al. performed transesophageal echocardiography using the GE Vingmed E9-series, but measured SV directly and used thermodilution with a pulmonary artery catheter as a reference method [30]. The study reported a bias of $-$1.2 mL, or $-$1.9%. Suehiro et al. also calculated an adjusted percentage error based on the standard deviation in differences between measurement methods of 20%.

For age calculations only years of birth were available, and such age is uncertain by up to 1 year, which for the average age in this population corresponds to 1.8%. For weight and height we assume only 0.1 $\text{kg}$ and 1 $\text{cm}$, i.e., approximately 0.1% and 0.6%, respectively. The estimated uncertainties can be seen in Table 11.

**Table 11:** Some estimated uncertainty levels in variables used in regression based on literature sources and estimates

Quantity	Estimated uncertainty (%)
ABPM SBP/DBP	~ 0.7
$\text{VO2max}$	~ 2.9
SV (4D)	~ 20
Age	~ 1.8
Height	~ 0.6
Weight	~ 0.1

SV, stroke volume; ABPM, ambulatory blood pressure monitoring; SBP, systolic blood pressure; DBP, diastolic blood pressure

### Models

**Fig. 5:**
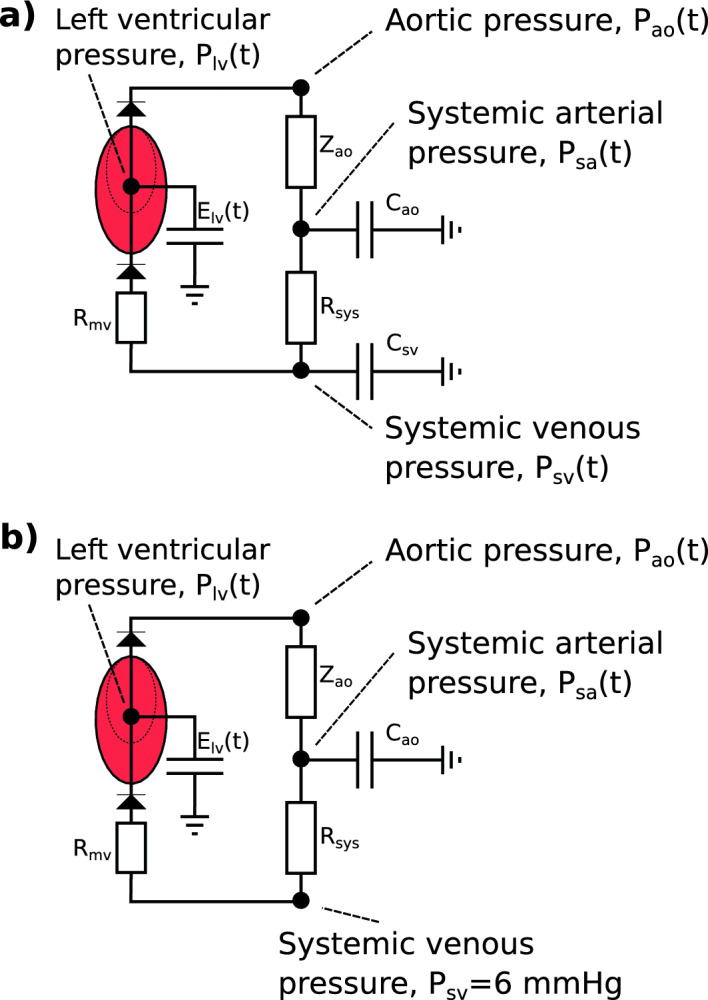
**a** The closed-loop, lumped-parameter model of the left ventricle, systemic arteries, and veins.** b** The open-loop lumped-parameter model of the left ventricle and systemic arteries. The circuit equivalent formulation of the models are depicted with the pressures and most of the mechanical parameters used to describe the systemic circulation. The venous compartment is volumeless and only partially described in the open-loop model. Pressures *P*, denote pressures in different parts of the cardiovascular system. Subscripts are as indicated by the figure text. $E_{\text{lv}}$ indicates the left-ventricular elastance function. $Z_{\text{ao}}$: characteristic aortic impedance, $C_{\text{ao}}$: systemic arterial compliance, $R_{\text{sys}}$: total peripheral resistance, $R_{\text{mv}}$: mitral valve resistance. Taken from Bjørdalsbakke et al. [17] under a CC-BY$-$4.0 License

**Fig. 6:**
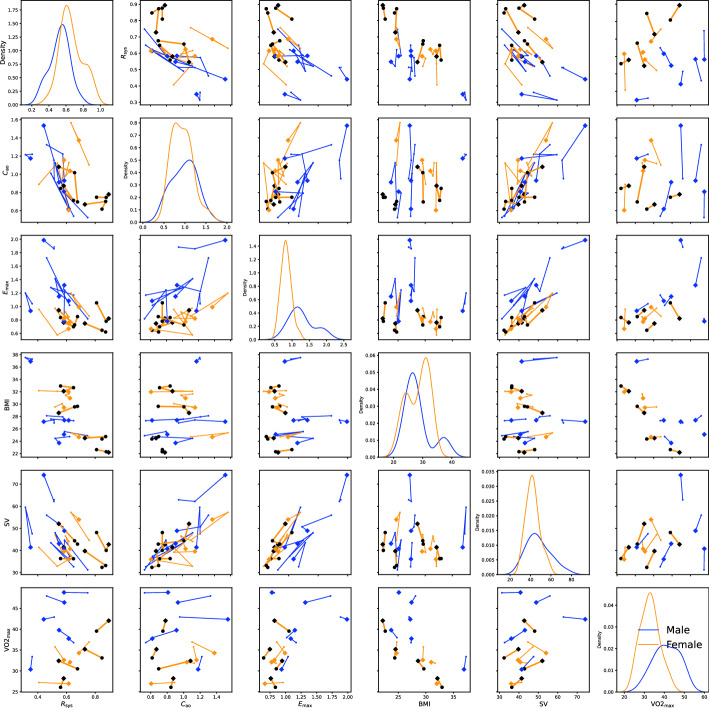
Pairplots of variables used in the regression analysis. The parameter values are optimized for the open-loop model. The participants with $\text{VO2}_{\text{max}}$ changes in the top quartile are marked by black markers. The final measurement day is marked by a diamond. The color encoding indicates sex

**Fig. 7:**
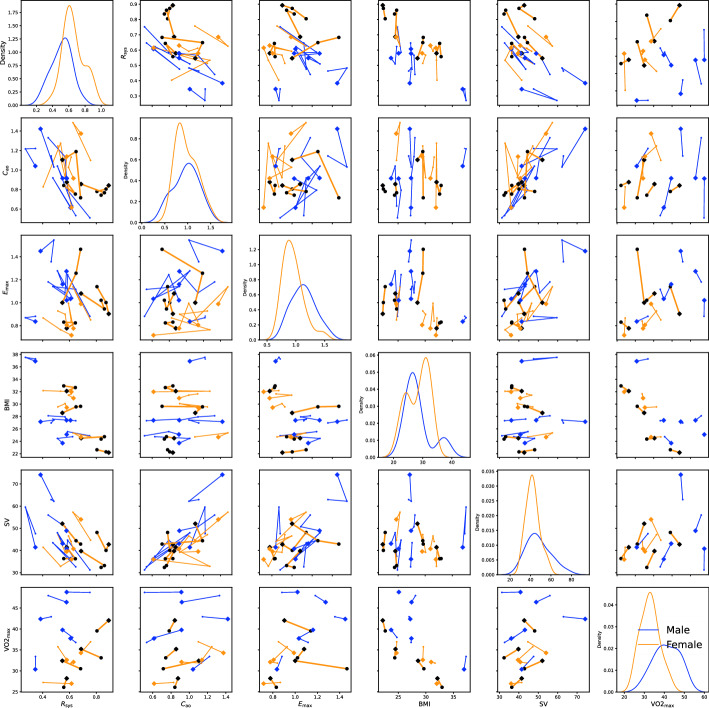
Pairplots of variables used in the regression analysis. The parameter values are optimized for the closed-loop model. The participants with $\text{VO2}_{\text{max}}$ changes in the top quartile are marked by black markers. The final measurement day is marked by a diamond. The color encoding indicates sex

**Fig. 8:**
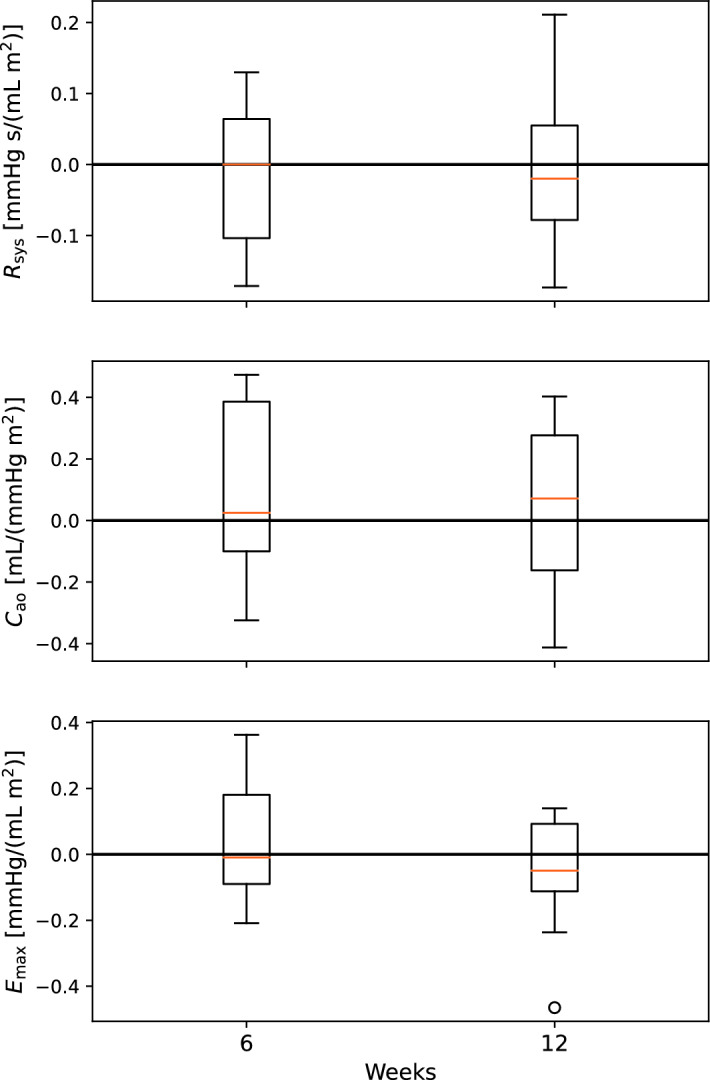
The figure presents the quartiles with wiskers for the changes in parameters relative to the parameters estimated at baseline, or on week 0. These results are made using the carotid pressure waveform and the closed-loop model

**Fig. 9:**
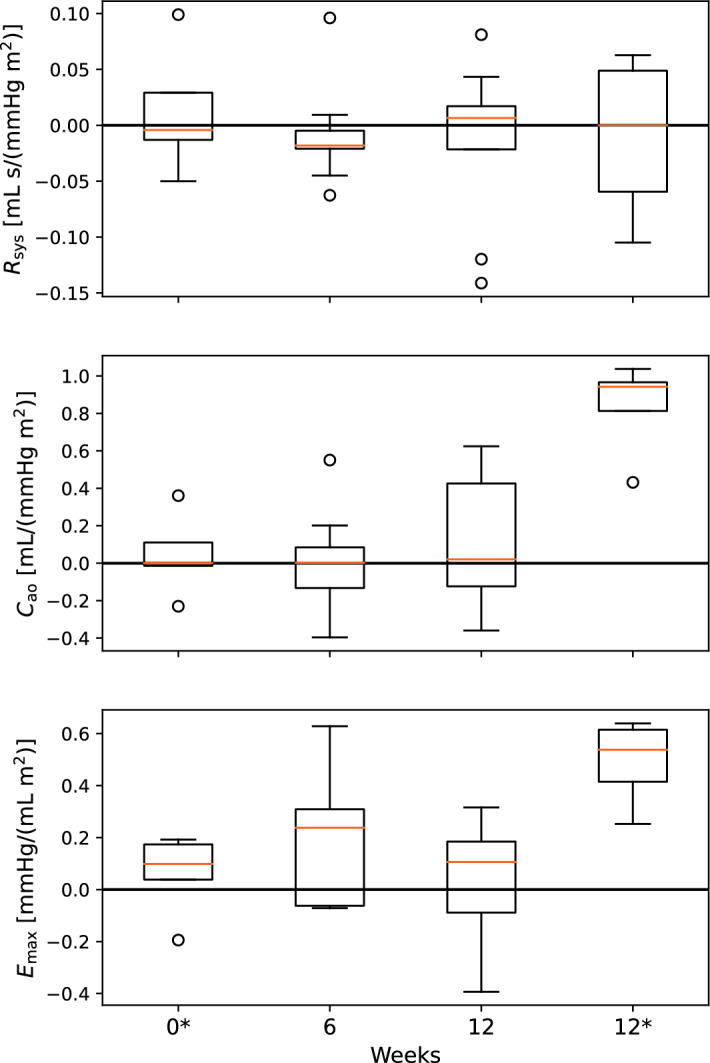
The figure presents the quartiles with whiskers for the changes in parameters relative to the parameters estimated at baseline, or on week 0. These results are made using the finger pressure waveform and the closed-loop model. The asterisk * indicates that the change is for parameters made 24-h post-CPET for the indicated week number

The aim of using physics-based models in this study is twofold. The first is to use limited hemodynamic data to try to estimate various mechanical parameters by the way the model constrict parameters to obey physical relationships in the circulatory system. Secondly, upon the successful personalization of a model, by parameters that have a physical interpretation and are informative about the individual’s CV health, the model may be subjected to stimuli which would give a prediction of how an individual may respond to exercise or other conditions or disease. The output quantities of interest, as investigated in this paper, to be produced by the models, are the aortic blood pressure ($P_{\text{ao}}$) and flow waveforms ($Q_{\text{lvao}}$). This includes the derived quantities of systolic and diastolic blood pressure, as well as ventricular volumes by way of stroke volume. The models used have been presented in detail previously by Bjørdalsbakke et al. [17, 23]. The closed-loop model is based on previous models by Smith et al., Segers et al., and Bovendeerd et al. [31–33]. The open-loop model is equivalent to the model presented by Stergiopulos et al. [34]. The models are depicted in Fig. 5, and the model parameters which are chosen for personalization are given in Table 12. Both models are investigated in this paper since they describe mainly the same hemodynamics with exception of the venous compartment and ventricular filling. They also have varying amounts of potentially personalizable parameters, which may interact during optimization and cause different parameter estimates between models. Initial parameter guesses are sampled from ranges as described previously [17]. The quantities of interest predicted by the model are mainly the stroke volume, and aortic pressure waveform, from which all pressure measurements, which are approximated to be equivalent to the corresponding brachial and finger measurements for this simplified model, are derived. Equivalently, the aortic blood flow produced by the model is approximated to be equivalent to the measured LVOT flow.

**Table 12:** The closed-loop model parameters are listed with their corresponding symbols and units

Symbol	Description	Units
$C_{ao}$	Systemic arterial compliance	$\mathrm {\frac{mL}{mmHg}}$
$C_{sv}$	Systemic venous compliance	$\mathrm {\frac{mL}{mmHg}}$
$E_{max}$	Maximal left ventricular elastance	$\mathrm {\frac{mmHg}{mL}}$
$E_{min}$	Minimal left ventricular elastance	$\mathrm {\frac{mmHg}{mL}}$
$R_{mv}$	Mitral valve resistance	$\mathrm {\frac{mmHg \ s}{mL}}$
$R_{sys}$	Total peripheral resistance	$\mathrm {\frac{mmHg \ s}{mL}}$
*T*	Heart period	s
$t_{peak}$	Time of peak ventricular elastance	s
$V_{tot}$	Total stressed blood volume	mL
$Z_{ao}$	Characteristic impedance of the aorta	$\mathrm {\frac{mmHg \ s}{mL}}$

The same parameters are used to describe the open-loop model except for $C_{sv}$ and $V_{tot}$

Taken from Bjørdalsbakke et al. [17] under a CC-BY–4.0 license

#### Model output and parameter estimation

The models were formulated as a set of differential equations where a 4th order Runge–Kutta scheme implemented in SciPy [35]. Model outputs are denoted as time-dependent signals $y(t, \varvec{\theta })$, where $\varvec{\theta }$ is a vector of mechanical model parameters.

Parameters are estimated by an ensemble of local optimizations, where the aortic flow and pressure waveforms with additional weight for systolic and diastolic values are included in the cost function. Hence, the parameter estimates have some inherent variability stemming from the optimization method, in addition to the intra-individual variability over time. The model was optimized by minimizing the following cost function:

1 $$\begin{aligned} J(\varvec{\theta })&= \sum _i^N \left( \frac{P^m_{\text{ao},i} - P_{\text{ao},i}}{K_\text{p}} \right) ^2 + \sum _i^N \left( \frac{Q^m_{\text{ao},i} - Q_{\text{ao},i}}{K_\text{q}} \right) ^2 \\&+ \frac{7.5^2 N^2}{40^2} \left[ \left( \frac{P^m_{\text{sys}} - P_{\text{sys}}}{K_{\text{p,sys}}} \right) ^2 + \left( \frac{P^m_{\text{dia}} - P_{\text{dia}}}{K_{\text{p,dia}}} \right) ^2 \right] \\&+ \frac{7.5^2 N^2}{40^2} \left[ \left( \frac{\text{SV}^m-\text{SV}}{K_{\text{SV}}} \right) ^2 + \frac{1}{9} \left( \frac{6.0 - \text{MVP}}{K_{\text{MVP}}} \right) ^2 \right] . \end{aligned}$$

Here, the $\text{m}$ superscript indicates a measurement while the corresponding measures are model predictions. *N* is the number of time points in the waveform sample, $P_{\text{sys}}$ and $P_{\text{dia}}$ are the systolic and diastolic values of the pressure waveform $P_{\text{ao}}$, while the aortic flow waveform is denoted as $Q_{\text{lvao}}$. The final term constrains the mean venous pressure ($\text{MVP}$). All constants *K* are scaling constants with a magnitude similar to a reference level for the different measurement types. For further details, settings, and bounds for the optimization routine see Bjørdalsbakke et al. [17]. The cost function was identical for both CV models formulations, except for the final venous pressure term, which was not applicable to the open-loop model.

The variability for parameters estimated for a single participant at a given point in time was found in previous work to be smaller than for the interpersonal parameter variability for the study population, and smaller than the intra-individual variability over all measurement days in a majority of cases. Further details on implementation, optimization and waveform processing can be found in Bjørdalsbakke et al. [17]. The parameters were normalized by body surface area (BSA), defined as $\text{BSA} = \sqrt{\frac{\text{height} \cdot \text{weight}}{3600}}$ [36]. BSA has units of m$^2$. This is to account for inter-individual variation in parameters that are known or can be assumed to be dependent upon such factors.

### Parameter and statistical analysis

In addition to the parameter estimates optimized to the model itself, we computed estimates based on conventional estimation methods for $C_{\text{ao}}$ and $R_{\text{sys}}$ by the formulas:

2 $$C_{{{\text{ao}}}}^{m} = \frac{{{\text{PP}}}}{{{\text{SV}}}},$$

and

3 $$R_{{{\text{sys}}}}^{m} = \frac{{{\text{MAP}}}}{{{\text{CO}}}}$$

For these equations $\text{PP}$ denotes pulse pressure, $\text{SV}$ is stroke volume, $\text{CO}$ is cardiac output, and $\text{MAP}$ is the mean arterial pressure as computed by averaging the carotid or finger pressure waveform calibrated by brachial blood pressure. Here, $\text{PP}$ is defined as the difference between systolic and diastolic brachial blood pressure.

The end-systolic pressure–volume relation (ESPVR) is often approximated to be linear [37]. Estimation methods based on single heart beats using ventricular data have been proposed [38, 39]. However, we do not use ventricular volumes in this investigation, so therefore we use another simpler method to estimate the maximal left-ventricular elastance by neglecting the volume axis intercept for the ESPVR (V$_{\text{d}}$) as follows:

4 $$E_{{max}}^{m} \approx \frac{{{\text{ESP}}}}{{{\text{ESV}}}} \approx \frac{{P_{{{\text{br,sys}}}} }}{{{\text{ESV}}}}.$$

Here, $\text{ESP}$ denotes left-ventricular end-systolic pressure, and $P_{\text{br,sys}}$ is the brachial systolic pressure. This estimate carries some additional uncertainty due to this measure being load dependent [37]. This means that the elastance estimated in this way changes with changed afterload of the heart, while the slope of the end-systolic pressure–volume relation in the normal operating range is normally load independent.

Equations (2, 3 and 4) allowed us to compute the changes in these parameters for comparison to changes estimated using model-optimized parameters. Estimates made by these equations will be referred to as “conventional estimates”.

#### Regression analysis

Ordinary Linear Regression analysis as implemented in the Python library “statsmodels” [40], and correlation analysis performed through by the “Pingouin” Python library were the main employed statistical tools for this exploratory analysis.

Ross et al. have suggested research designs and statistical methods to model exercise response variability to changes in CRF for different study designs [41]. For our analysis, we instead include CRF as a predictor to explain changes in model parameters. Ross et al. recommend using linear mixed-effects models for trials with repeated measurements over time, but without a control groups. We consequently look at the personalized parameters where the parameter for an individual *i* is given as:

5 $$\begin{aligned} \theta _i = \mu + \alpha _i + \varepsilon _i. \end{aligned}$$

Further, $\mu$ indicates the population average parameter, including both the intercept, and common regressor coefficients estimated for the population. Simultaneously, $\alpha _i$ is the personal deviation from the average, which includes permanent effects such as sex, and transient effects such as age and lifestyle (diet, activity level, etc.), and we only allowed personal deviations in the model intercept for our analysis. $\varepsilon _i$ indicates all sources of random error, which includes measurement errors, and error from the estimation procedure, but also short-term day-to-day variation. Although the model structure for the dynamic CV model may be simple, the behavior of state variables and model parameters may be complex and depend on data quality, noise, and the model structure itself. However, we are interested to see whether the data hold patterns which may be linked to how the parameters are influenced by fitness level, as they change over the study period. In order to understand some of these relationships better, we apply ordinary linear regression and linear mixed modeling in the context of Eq.(5), to relate measurements, and exercise stimuli to the estimated model parameters and their changes after the intervention period. The linear mixed model fits a model for estimated parameters at day 1, 2, and 3 of the measurement period to hemodynamic and fitness measurements made at corresponding time points, while accounting for individual variability in this relationship. Ordinary regression is applied to investigate the explanatory variables of the direct change in parameters. Our models are built in various configurations using age, sex, BMI, SV, $\text{VO2max}$, and changes in the last three. The letters A–D indicate different models with different choices of the listed regressors used to predict the chosen parameter value or change. A is the baseline model (including age, sex, and BMI), B includes $\text{VO2max}$, C includes SV, and D includes all listed predictors. An overview of the model configurations can be seen in Table 13.

**Table 13:** The different regression cases and what variables are used as dependent and independent variables

Case	Dependent variable	Independent variables
A	$R_{\text{sys}}$/$C_{\text{ao}}$/$E_{\text{max}}$	Age, sex, BMI
B	$R_{\text{sys}}$/$C_{\text{ao}}$/$E_{\text{max}}$	Age, sex, BMI, $\text{VO2max}$
C	$R_{\text{sys}}$/$C_{\text{ao}}$/$E_{\text{max}}$	Age, sex, BMI, SV
D	$R_{\text{sys}}$/$C_{\text{ao}}$/$E_{\text{max}}$	Age, sex, BMI, $\text{VO2max}$, SV

This applies both for the ordinary linear regression models, and the linear mixed-effects models

To investigate regression to the mean, we tested whether the value on the last measurement day of an individual’s parameters highly correlated to the deviation of the individual’s average value ($\theta _{\text{pers,avg}}$) from the population average parameter value over all measurement days ($\theta _{\text{pop}}$). The individual average was computed using only the first two measurement days. A high adjusted $r^2$ would indicate how much variance is explained in the final parameter value by the personal average variability, and indicate a clustering about a personal mean. The clinical trial was uncontrolled in terms of parameter changes and non-exercising participants. Therefore, we needed to test whether it was likely that calculated changes were due to extreme observations of random effects, or if they were influenced by the exercise stimulus or other causes. Additionally, the change of the parameter value between measurement day 1 and 3 was tested by whether it would correlate highly to the difference between the initial parameter value and the population value. In this case, a high negative correlation would indicate regression to the population mean. We refer to these two investigations of regression to the mean as the “regression to the mean analyses”.

Next, for the second part of the regression analysis focusing on other covariates than parameter values, we investigated whether the level of or change in CRF could explain variability in the parameters and parameter changes. This was assessed by comparing unexplained (estimated residual variance) and explained variance (adjusted $r^2$) for regression models. We designed models for determining either the parameter change over all 12 weeks, or the parameter development over all 12 weeks including individual variation. We would then assess whether any added parameters contributed to explaining more of the observed variability. For prediction of changes, the changes from day 1 to 3 were analyzed for the parameters $E_{\text{max}}$, $C_{\text{ao}}$, and $R_{\text{sys}}$. The change between measurement day *j* and *i* is denoted as $\Delta _{i,j}$. For all regression models, the dependent variables are Z-score standardized. The regressors for the linear mixed effects models are grand mean centered for the standardization.

## Data Availability

The datasets generated and/or analyzed during the current study are not publicly available to protect participant confidentiality.

## Participant characteristics

Tables 14 and 15 present additional statistics about the participants in the different subgroups based on different blood pressure waveforms.

**Table 14:** Baseline characteristics of the study population included with carotid pressure waveforms

	*n* = 14
Height	174.1 ± 8.9 cm
Weight	86.3 ± 15.5 kg
BSA	2.0 ± 0.2 m$^2$
BMI	28.4 ± 4.1 kg/m$^2$
Age	56.5 ± 3.7 years
Sex M/F	6/8
SBP	136.0 ± 7.4 mmHg
DBP	84.8 ± 6.0 mmHg
$\text{VO2}_{\text{max}}$	36.4 ± 6.8 mL/(kg min)
24h ABPM awake SBP	137.1 ± 10.5 mmHg
24h ABPM awake DBP	82.9 ± 7.0 mmHg
SV (4D)	88.7 ± 21.5 mL
SV LVOT	85.7 ± 20.1 mL

BSA: body surface area; BMI: body mass index; ABPM: ambulatory blood pressure monitoring; SV: stroke volume; LVOT: left-ventricular outflow tract

SBP and DBP signifies systolic and diastolic office blood pressure measurement

4D refers to 3D measurement averaged over time

**Table 15:** Baseline characteristics of the study population included with finger pressure waveforms

	*n* = 9
Height	171.2 ± 9.8 cm
Weight	79.2 ± 11.6 kg
BSA	1.9 ± 0.2 m$^2$
BMI	27.0 ± 3.4 kg/m$^2$
Age	57.3 ± 3.3 years
Sex M/F	3/6
SBP	138.6 ± 9.0 mmHg
DBP	85.3 ± 5.5 mmHg
$\text{VO2}_{\text{max}}$	34.8 ± 8.3 mL/(kg min)
24h ABPM awake SBP	133.7 ± 7.3 mmHg
24h ABPM awake DBP	81.8 ± 5.8 mmHg
SV (4D)	73.3 ± 11.2 mL
SV LVOT	75.6 ± 15.8 mL

BSA: body surface area; BMI: body mass index; ABPM: ambulatory blood pressure monitoring; SV: stroke volume; LVOT: left-ventricular outflow tract

SBP and DBP signifies systolic and diastolic office blood pressure measurement

4D refers to 3D measurement averaged over time

## Closed-loop model results

The appended Tables 16, 17, 18, and 19 show results for the closed-loop cardiovascular model.

**Table 16:** Maximal, minimal, average and average absolute changes for total peripheral resistance ($R_{\text{sys}}$), systemic arterial compliance ($C_{\text{ao}}$), and maximal left-ventricular elastance ($E_{\text{max}}$)

Parameter	Wave-form	Estimation method	Maximal abs. change	Minimal abs. change	Maximal change	Minimal change	Mean change	Mean abs. change	Units
$R_{\text{sys}}$	C	Model	0.211	0.001	0.211	− 0.174	− 0.009(0.097)	0.081(0.055)	$\frac{\mathrm {mmHg \ s}}{\text{mL}\mathrm {m^2}}$
$C_{\text{ao}}$	C	Model	0.660	0.004	0.473	− 0.660	0.037(0.261)	0.220(0.151)	$\frac{\text{mL}}{\text{mmHg}\mathrm {m^2}}$
$E_{\text{max}}$	C	Model	0.465	0.007	0.362	− 0.465	− 0.027(0.161)	0.133(0.094)	$\frac{\text{mmHg}}{\text{mL}\mathrm {m^2}}$
$R_{\text{sys}}$	C	Conv.	0.213	0.000	0.213	− 0.203	− 0.011(0.111)	0.091(0.064)	$\frac{\mathrm {mmHg \ s}}{\text{mL}\mathrm {m^2}}$
$C_{\text{ao}}$	C	Conv.	0.324	0.001	0.265	− 0.324	0.016(0.145)	0.122(0.080)	$\frac{\text{mL}}{\text{mmHg}\mathrm {m^2}}$
$E_{\text{max}}$	C	Conv.	0.793	0.004	0.504	− 0.793	− 0.074(0.246)	0.188(0.176)	$\frac{\text{mmHg}}{\text{mL}\mathrm {m^2}}$
$R_{\text{sys}}$	F	Model	0.144	0.003	0.144	− 0.141	− 0.010(0.064)	0.047(0.045)	$\frac{\mathrm {mmHg \ s}}{\text{mL}\mathrm {m^2}}$
$C_{\text{ao}}$	F	Model	0.625	0.001	0.624	− 0.398	0.077(0.282)	0.207(0.207)	$\frac{\text{mL}}{\text{mmHg}\mathrm {m^2}}$
$E_{\text{max}}$	F	Model	0.631	0.006	0.628	− 0.631	0.027(0.281)	0.234(0.159)	$\frac{\text{mmHg}}{\text{mL}\mathrm {m^2}}$
$R_{\text{sys}}$	F	Conv.	0.163	0.002	0.157	− 0.163	− 0.019(0.072)	0.055(0.050)	$\frac{\mathrm {mmHg \ s}}{\text{mL}\mathrm {m^2}}$
$C_{\text{ao}}$	F	Conv.	0.243	0.001	0.243	− 0.113	0.065(0.102)	0.098(0.070)	$\frac{\text{mL}}{\text{mmHg}\mathrm {m^2}}$
$E_{\text{max}}$	F	Conv.	0.793	0.019	0.161	− 0.793	− 0.175(0.210)	0.207(0.179)	$\frac{\text{mmHg}}{\text{mL}\mathrm {m^2}}$

Results are given for parameter changes produced by closed-loop model optimization and by computation using conventional equations, based on both carotid (C) and finger (F) pressure waveforms

Maximal and minimal changes are given unsigned, and can be any changes between the first, second and third measurement days

The carotid measurements describe 14 participants with carotid pressure measurements, while the finger pressure includes 9 participants with synchronized flow and pressure

**Table 17:** Correlation statistics for parameter changes from total peripheral resistance ($R_{\text{sys}}$), systemic arterial compliance ($C_{\text{ao}}$), and maximal left-ventricular elastance ($E_{\text{max}}$)

Parameter	Wave-form	Temporal change (meas. day)	*r*	*p* value	CI95%
$R_{\text{sys}}$	C	1–2	0.994	$<10^{-3}$	[0.98, 1.00]
$R_{\text{sys}}$	C	1–3	0.995	$<10^{-3}$	[0.98, 1.00]
$R_{\text{sys}}$	C	2–3	0.990	$<10^{-3}$	[0.97, 1.00]
$R_{\text{sys}}$	C	All	0.993	$<10^{-3}$	[0.99, 1.00]
$C_{\text{ao}}$	C	1–2	0.594	0.025	[0.09, 0.86]
$C_{\text{ao}}$	C	1–3	0.816	$<10^{-3}$	[0.50, 0.94]
$C_{\text{ao}}$	C	2–3	0.594	0.025	[0.09, 0.85]
$C_{\text{ao}}$	C	All	0.674	$<10^{-3}$	[0.47, 0.81]
$E_{\text{max}}$	C	1–2	− 0.286	0.343	[− 0.72, 0.31]
$E_{\text{max}}$	C	1–3	− 0.631	0.021	[− 0.88, − 0.12]
$E_{\text{max}}$	C	2–3	− 0.222	0.446	[− 0.67, 0.35]
$E_{\text{max}}$	C	All	− 0.391	0.013	[− 0.63, -0.09]
$R_{\text{sys}}$	F	1–2	0.978	$<10^{-3}$	[0.90, 1.00]
$R_{\text{sys}}$	F	1–3	0.991	$<10^{-3}$	[0.95, 1.00]
$R_{\text{sys}}$	F	2–3	0.995	$<10^{-3}$	[0.98, 1.00]
$R_{\text{sys}}$	F	All	0.990	$<10^{-3}$	[0.98, 1.00]
$C_{\text{ao}}$	F	1–2	− 0.124	0.750	[− 0.73, 0.59]
$C_{\text{ao}}$	F	1–3	0.320	0.402	[− 0.44, 0.81]
$C_{\text{ao}}$	F	2–3	0.525	0.147	[− 0.21, 0.88]
$C_{\text{ao}}$	F	All	0.215	0.281	[− 0.18, 0.55]
$E_{\text{max}}$	F	1–2	− 0.192	0.650	[− 0.59, 0.79 ]
$E_{\text{max}}$	F	1–3	− 0.321	0.438	[− 0.84, 0.50]
$E_{\text{max}}$	F	2–3	− 0.418	0.263	[− 0.85, 0.34]
$E_{\text{max}}$	F	All	− 0.327	0.110	[− 0.64, 0.08]

Results are given for correlations between parameter changes produced by closed-loop model optimization and by computation using conventional techniques, based on both carotid (C) and finger (F) pressure waveforms

**Table 18:** Average and average absolute parameter changes over different periods throughout the study period, describing the parameter change from measurements made before a CPET and the day after

Parameter	Estimation change	Temporal change	Mean abs. change	Mean change
$R_{\text{sys}}$	Model	CPET week 0	0.046(0.040)	0.018(0.063)
$R_{\text{sys}}$	Model	CPET week 12	0.059(0.020)	0.026(0.063)
$R_{\text{sys}}$	Model	Pre-CPET week 0-12	0.047(0.063)	− 0.036(0.070)
$R_{\text{sys}}$	Conv.	CPET week 0	0.052(0.043)	0.016(0.072)
$R_{\text{sys}}$	Conv.	CPET week 12	0.064(0.022)	0.035(0.065)
$R_{\text{sys}}$	Conv.	Pre-CPET week 0-12	0.055(0.066)	− 0.053(0.067)
$C_{\text{ao}}$	Model	CPET week 0	0.121(0.167)	0.114(0.173)
$C_{\text{ao}}$	Model	CPET week 12	0.183(0.356)	− 0.179(0.359)
$C_{\text{ao}}$	Model	Pre-CPET week 0-12	0.312(0.348)	0.312(0.348)
$C_{\text{ao}}$	Conv.	CPET week 0	0.072(0.071)	0.068(0.076)
$C_{\text{ao}}$	Conv.	CPET week 12	0.031(0.024)	− 0.003(0.044)
$C_{\text{ao}}$	Conv.	Pre-CPET week 0-12	0.127(0.069)	0.127(0.069)
$E_{\text{max}}$	Model	CPET week 0	0.126(0.071)	0.126(0.071)
$E_{\text{max}}$	Model	CPET week 12	0.177(0.116)	− 0.017(0.234)
$E_{\text{max}}$	Model	Pre-CPET week 0-12	0.113(0.078)	0.113(0.078)
$E_{\text{max}}$	Conv.	CPET week 0	0.082(0.090)	− 0.082(-0.090)
$E_{\text{max}}$	Conv.	CPET week 12	0.070(0.040)	0.070(0.040)
$E_{\text{max}}$	Conv.	Pre-CPET week 0-12	0.281(0.078)	− 0.281(0.078)

Standard deviations are given in parentheses

Pre-CPET denotes parameter changes calculated between the first and final measurement day based on measurements made prior to the CPET

Results are given for parameter changes produced by closed-loop model optimization and by computation using conventional techniques, based on the finger pressure waveforms

**Table 19:** Ordinary linear regression models for changed model parameters based on the difference between the individual average parameter values over the two first measurement days (subscript: avg) or the baseline parameter value and the population average (subscript: pop)

DV:	$R_{\text{sys,3}}$	$C_{\text{ao,3}}$	$E_{\text{max,3}}$	$\Delta _{1,3} R_{\text{sys}}$	$\Delta _{1,3} C_{\text{ao}}$	$\Delta _{1,3} E_{\text{max}}$
Intercept	0.590*	0.967*	0.988*	− 0.013	0.056	− 0.041
$R_{\text{sys,avg}}$-$R_{\text{sys,pop}}$	0.102*					
$C_{\text{ao,avg}}$-$C_{\text{ao,pop}}$		0.112*				
$E_{\text{max,avg}}$-$E_{\text{max,pop}}$			0.164*			
$R_{\text{sys,1}}$-$R_{\text{sys,pop}}$				− 0.061*		
$C_{\text{ao,1}}$-$C_{\text{ao,pop}}$					− 0.149*	
$E_{\text{max,1}}$-$E_{\text{max,pop}}$						− 0.063
Adj. $r^2$	0.637	0.231	0.644	0.265	0.319	0.076
N	14	14	14	14	14	14
F-statistic	23.81	4.911	24.47	5.682	7.095	2.067
F-test, p-value	0.000*	0.047*	0.000*	0.035*	0.021*	0.176

The model parameters are optimized for the closed-loop model using the carotid pressure waveform

Indices, 1–3 indicate measurement day

DV: dependent variable

## Pairplots

Figures 6 and 7 show the pairplots for scatterplots between different variables applied in the regression analysis as well as the participants included in the case study. The changes of parameters compared to the baseline estimates using the closed-loop CV model can be seen in Figs. 8 and 9.
